# Systemic modeling myeloma-osteoclast interactions under normoxic/hypoxic condition using a novel computational approach

**DOI:** 10.1038/srep13291

**Published:** 2015-08-18

**Authors:** Zhiwei Ji, Dan Wu, Weiling Zhao, Huiming Peng, Shengjie Zhao, Deshuang Huang, Xiaobo Zhou

**Affiliations:** 1 School of Electronics and Information Engineering, Tongji University, Shanghai, P.R. China 201804; 2 Division of Radiologic Sciences – Center for Bioinformatics and Systems Biology, Wake Forest School of Medicine, Medical Center Boulevard, Winston-Salem, NC, USA 27157

## Abstract

Interaction of myeloma cells with osteoclasts (OC) can enhance tumor cell expansion through activation of complex signaling transduction networks. Both cells reside in the bone marrow, a hypoxic niche. How OC-myeloma interaction in a hypoxic environment affects myeloma cell growth and their response to drug treatment is poorly understood. In this study, we **i**) cultured myeloma cells in the presence/absence of OCs under normoxia and hypoxia conditions and did protein profiling analysis using reverse phase protein array; **ii**) computationally developed an Integer Linear Programming approach to infer OC-mediated myeloma cell-specific signaling pathways under normoxic and hypoxic conditions. Our modeling analysis indicated that in the presence OCs, (1) cell growth-associated signaling pathways, PI3K/AKT and MEK/ERK, were activated and apoptotic regulatory proteins, BAX and BIM, down-regulated under normoxic condition; (2) β1 Integrin/FAK signaling pathway was activated in myeloma cells under hypoxic condition. Simulation of drug treatment effects by perturbing the inferred cell-specific pathways showed that targeting myeloma cells with the combination of PI3K and integrin inhibitors potentially (1) inhibited cell proliferation by reducing the expression/activation of NF-κB, S6, c-Myc, and c-Jun under normoxic condition; (2) blocked myeloma cell migration and invasion by reducing the expression of FAK and PKC under hypoxic condition.

Multiple myeloma (MM) is the second most common hematological malignancy and is characterized by the clonal expansion of plasma cells in the bone marrow^1^. Myeloma cells reside in the bone marrow (BM), which is composed of various stromal cells, including osteoclasts (OCs), osteoblasts, endothelial cells and fibroblasts, as well as immune cells^2^. Therefore, bone marrow niche is critical for myeloma cell proliferation, growth and migration through provision of survival signals and secretion of cytokines, chemokines and growth factors^3^,^4^. OCs are derived from bone marrow stem cells and play an important role in bone degeneration. Early studies have showed that OCs stimulated myeloma cell growth and survival via a cell-cell interaction^5^. However, the detailed mechanisms have not been well studied.

BM has long been accepted as a naturally hypoxic organ^6^. The spatial distribution of oxygen in BM is heterogeneous, thus, BM compartments contains different oxygen tensions^7^,^8^. The bone-BM interface is strongly hypoxic and vascular niche comparatively less hypoxic^1^. Hypoxia has been associated with an increased risk of metastasis and mortality in many human cancers^9^. Early studies have devoted to explore the molecular mechanisms underlying the effect of intratumoral hypoxia on cancer progression^10^. The molecular responses of myeloma cells in a hypoxia environment have been studied by several groups^11^,^12^. However, the impact of OCs-myeloma cell interactions on myeloma growth under hypoxic condition has not been explored. In this study, we developed a novel computational approach to model the effect of OCs on myeloma cell growth and revealed the relevant molecular mechanism.

Human myeloma cell line RPMI 8226 and primary OC cells were co-cultured under either normoxic or hypoxic condition and protein samples of RPMI 8226 cells collected at 5 h, 24 h and 48 h post-treatment. An integrated proteomic strategy of reverse phase protein arrays (RPPA) was applied to assess the changes in the signaling molecules associated with cell proliferation, apoptosis, migration, and adhesion. Based on our proteomics data and a prior set distribution of potential generic pathways, two generic signaling networks of myeloma cells were built manually for normoxic and hypoxic conditions. Then the time-series RPPA data were applied to the generic signaling networks to infer OCs-mediated myeloma-specific pathways.

Two major types of pathway inference approaches have been used to optimize cell-specific pathways from the proteomics data: ordinary differential equations (ODEs) modeling approaches^13^,^14^ and discrete modeling approaches^15^,^16^,^17^,^18^. Commonly, many parameters are needed in the ODEs modeling approaches to model the dynamics of signaling networks, however, the parameter estimation is very challenging when simulating large-scale networks with small samples^19^. Hence, ODE modeling approach is not flexible in determining the topology of signaling networks in this study. On the other hand, discrete modeling approaches include Boolean operation based approaches^16^,^18^ and Ternary operation approaches^17^. In Boolean operation based approaches, the status of a kinase were normalized as activated (“1”) or inactivated (“0”) for qualitatively analyzing large-scale signaling pathways. However, Boolean states used in these approaches are not sufficient enough to represent the variations of phosphor-signals under different conditions. In Melas’s discrete model, three possible states for signaling proteins were taken into account, including up-regulation (valued as “1”), down-regulation (“−1”), and no-change (“0”); and the pathway topologies under various perturbations were assumed to be the same. This approach could not be directly applied to solve our problem because the activation of signaling pathways in our study was involved in dynamic changes at different time points. Thus, we proposed to develop a time-series-data-driven Integer Linear Programming (simply called as dynamic ILP or DILP) approach to infer OCs-mediated myeloma-specific signaling pathways by detecting topology alterations of the signaling network at different times (See Fig. 1).

Our modeling analysis indicated that in the presence of OCs (1) the growth and proliferation-associated signaling pathways were activated, including PI3K/AKT and MEK/ERK, and apoptotic regulatory proteins, BAX and BIM, down-regulated under normoxic condition; (2) β1 Integrin/FAK signaling pathway was activated in myeloma cells under hypoxic condition. Analysis of specific pathway networks of myeloma cells provided an insight into the molecular mechanisms of myeloma cell survival and growth under normoxic and hypoxic conditions.

Based on the inferred myeloma-specific pathways, we simulated drug treatment effects by perturbing the inferred cell-specific pathways with PI3k and integrin inhibitors, simultaneously. The simulation results indicated that targeting myeloma cells with the combination of PI3K and integrin inhibitors potentially (1) inhibited cell proliferation by inhibiting the expression/activation of NF-κB, S6, c-Myc, and c-Jun under normoxic condition; (2) blocked myeloma cell migration and invasion by reducing the expression of FAK and PKC under hypoxic condition.

As a general tool for qualitative analysis of signaling pathways, the DILP-based discrete modeling approach is suited to effectively infer large-scale signaling pathways and predict qualitative behavior of the signal transduction system.

## Results

### RPMI 8226 myeloma cells are resistant to hypoxia when cocultured with OCs

OCs were generated from human peripheral blood mononuclear cells in the presence of RANKL and macrophage colony-stimulating factor. The formation of osteoclasts was demonstrated qualitatively by tartrate-resistant acid phosphatase staining as shown in the Supplementary Fig. S1differentiation rate of OCs was up to 90%. In order to determine the effect of OCs on myeloma cell growth, RPMI 8226 myeloma cells were cultured in the presence or absence of OCs up to 72 h. Cell proliferation was quantified by measuring dsDNA contents. As shown in Fig. 2A, RPMI 8226 cells co-cultured with OCs had an increased proliferation rate (1.5 fold higher) under normoxic condition, when compared with RPMI 8266 cells cultured without OCs. We then evaluated the effect of hypoxia on myeloma cell growth w/wo OCs. The average oxygen tension of bone marrow in MM patients was around 5% (ranged from 1–7%)^20^. 5% O_2_ was often used to simulate hypoxic condition *in vitro*^21^,^22^,^23^. Therefore, in our study, we used 5% O_2_ as a hypoxic condition and 21% O_2_ as a normoxic condition. Incubation of RPMI929 cells in the hypoxic condition led to a significant increase in the HIF1 expression at 24 h and 48 h post-treatment (Supplementary Fig. S2), when compared with the cells cultured in the normoxia incubator^20^. Under hypoxic condition, 37.6% of RPMI 8226 cells were survived in the presence of OC and 21.4% without OCs, indicating that OCs protected myeloma cells from hypoxia injury (Fig. 2B).

### RPPA data analysis

Our RPPA data were collected from four treatment conditions at three time points. The treatment groups included (1) myeloma cells co-cultured with OC under normoxia, (2) myeloma cells co-cultured with OC under hypoxia, (3) myeloma cells cultured without OC under normoxia, and (4) myeloma cells cultured without OC under hypoxia. Protein samples were harvested at 5 h, 24 h, and 48 h post-treatment. The RPPA dataset was divided into two segments. The first segment was used to infer myeloma-specific pathways under normoxic condition for understanding the impact of osteoclast-myeloma cell-cell interaction on myeloma growth. The fold change ratio (*ρ*) of each protein was calculated as myeloma co-cultured with OCs vs. without OCs under normoxia. The second segment was applied to infer myeloma-specific pathways under hypoxia for understanding the response of myeloma cells to hypoxia in presence or absence of OCs. We then calculated the ratios of fold changes for each protein, including myeloma co-cultured with OC under hypoxia against normoxia; myeloma without OC under hypoxia against normoxia. The significant up-regulated or down-regulated proteins were screened with the thresholds as *ρ* ≥ 1.2 or *ρ* ≤ 0.8 (Supplementary Fig. S3).

### Construction of generic pathway maps of myeloma cells for normoxic and hypoxic conditions

Based on the ratios of fold change for all the proteins in our RPPA dataset, we screened the differentially expressed proteins and imported them into IPA (http://www.ingenuity.com). Combining the top-ranked enriched pathways from IPA and the myeloma-related pathways reported in the literatures^24^, we manually built up two generic OC-mediated myeloma-related pathway maps for both of normoxic and hypoxic conditions (Fig. 3). The Figure 3A denotes the generic OC-mediated myeloma-related pathway map in normoxia. This map mainly associated with three cell functions: cell cycle regulation, cell proliferation, and apoptosis. The up-regulation of tumor suppressor p53 can arrest cell cycle to repair DNA-lesion^25^. NF-κB has been shown promoting cell survival, proliferation, and resistance to anticancer drugs^13^,^26^. AKT/mTOR/P70S6K pathway plays an important role in the cell growth and proliferation in multiple myeloma^27^. c-Jun, a transcription factor, promotes cell growth and proliferation in many cancer cells^28^. The pro-apoptotic proteins BAX and BIM both regulate the activation of the mitochondrial cell death pathway^29^,^30^. Figure 3B shows the generic OC-mediated myeloma-related pathway map in hypoxia. Except PI3K/AKT, MEK/ERK, and JNK pathways, integrin-FAK signaling pathways was presented in this map, which is associated with cell migration and invasion.

There are 31 nodes and 45 edges included in the pathway maps in Fig. 3A, and 31 nodes and 42 edges in Fig. 3B. Nodes in the pathway network represented signaling proteins with their discrete values 1,−1, or 0. These values represented the status of proteins and 1, −1, and 0 standed for “up-regulation”, “down-regulation”, and “no-change”, respectively. There are two types of edges in the signaling pathways: activation reaction (“→”) and inhibitory reaction (“
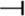
”). They were encoded by integer variables, which had discrete values 1 (activation) or −1 (inhibitory)^16^. The state of each reaction at certain time point is represented with logical values (“occur”(0) or “does not occur”(1)). In this study, we summarized five types of linking patterns of signaling proteins which were common in most of signaling network topologies (See Methods). In our DILP model of signaling network, the states of nodes and connected edges were constrained with the ***state consistent rules*** (see Supplementary Text S1 example, there was an “activation” edge from PDK1 to AKT in the generic pathway in Fig. 3A. Our developed linear constraints restrict that the states of PDK1 and AKT would be positive related if the reaction (PDK1 → AKT) occurred at certain time point. In addition, all the edges, connected to the same protein, were considered as independent. In another word, a downstream protein could be regulated by several upstream kinases simultaneously and its status was determined by all of its upstream proteins. The inference for states of all the nodes and edges was implemented with a set of constraints in our DILP model. The details regarding these constraints were described in Supplementary Text S2.

### Inference of OC-mediated myeloma-specific pathways by DILP

To infer cell-specific pathways based on the generic pathway map constructed above, we minimized the differences between measured and predicted values, as well as the complexity of inferred signaling network topology. To solve this multi-objective optimization problem, we developed the DILP model of the generic pathway map and obtained the cell-specific pathways by optimizing formula (3) in Methods (also see Supplementary Text S2, S3, and S4). The DILP model was solved with the MATLAB optimization toolbox Gurobi 5.6^31^, which guarantees minimal differences between proteomics data and predicted data, as well as the simplification of signaling network topology.

The inferred myeloma cell-specific pathway networks in the presence of OCs under normoxic/ hypoxic conditions are shown in Figs 4 and 5. Comparing with the signaling network shown in Fig. 3, several redundant edges were removed from the generic pathway networks due to the inconsistencies between these links and experimental measurements of connected nodes. Figure 4 represents the myeloma-specific pathway network in the presence of OCs under normoxic condition, in which the pathways of AMPK/mTOR and p53/p21 were removed from the generic pathway network, as shown in Fig. 3A. The fitting precision (defined in Supplementary Text S3) of the inferred cell-specific pathways in normoxia was 84.72%. The downstream proteins shown in Fig. 4 were mainly involved in two functional modules: the down-regulated cell apoptosis, and up-regulated cell proliferation. Our prediction of DILP model showed that three receptors, VEGFR, EGFR, and ERBB2/ERBB3, were up-regulated, which play an important role in stimulation of cell proliferation and inhibition of apoptosis^32^. PI3k/AKT and its downstream signaling molecules NF-κB and p70S6K, were activated at 24 h and 48 h following co-incubation with OCs. Particularly, the activation of NF-κB confers drug resistance in MM^24^. Transcription factors c-Jun and c-Myc were up-regulated at 5 h and/or 24 h. Anti-apoptosis machinery was activated to protect cells from apoptosis, including increased expression of cell survival proteins AKT and FOXO3, and reduced levels of apoptosis proteins of caspase 8, BAX, and BIM.

Figure 5A,B represent the inferred myeloma-specific pathways in the presence vs. absence OC under hypoxia. The phenotype-related functional modules were also highlighted in Fig. 5. All these pathways were inferred by fitting the generic pathway map with two sets of calculated ratios of corresponding proteins (myeloma without OC in the presence of hypoxia relative to normoxia; myeloma with OC in the presence of hypoxia relative to normoxia). The fitting precision of these two inferred myeloma-specific pathway networks in hypoxia is about 89.33%. Comparing with the signaling network shown in Fig. 3B, some edges were removed in the inferred networks. Comparing Fig. 5B to A, we can clearly see some of similarities between two of them. The protein level of NF-κB and S6 were reduced and E-cadherin and Yes-associated protein (Yap) up-regulated in both networks. Early studies showed overexpression of E-cadherin hinders tumor growth by suppressing PI3K/AKT signaling via B-catenin^33^. Yap plays a pivotal role in tumor suppression by restricting proliferation^34^.

Several proteins were up-regulated in the myeloma cells co-cultured with OCs under hypoxia condition as shown in Fig. 5B, including fibronectin, integrin, EGFR, PKC, FAK, BCL2 and P53. Soluble fibronectin can bind to integrin and induce the activation of FAK. Activation of FAK has been linked to cancer cell migration and invasion^32^. EGFR signaling plays an important role in cell proliferation and migration through downstream PKC pathway. Increased expression of p53 and p21 in Fig. 5B suggested that an induction of cell cycle arrest to allow cells repair their DNA damage^35^. BCL2 is a well-known anti-apoptotic protein. Increased expression of BCL2 may also contribute the attenuated hypoxia-induced cell death. The predicted values of key proteins at three time points were detailedly represented in Supplementary Fig. S4-S5.

### Experimental validation of signaling proteins

To validate the changes of signaling molecules in myeloma cells cultured with/without OCs under normoxic condition, myeloma cells were harvested after culturing for 24 h in the presence or absence of OCs. Western blot were processed to determine the key signaling proteins in the specific pathway of myeloma cells that was inferred above. As shown in Fig. 6, the phosphorylated protein levels of AKT, MEK, and ERK were increased in the RPMI 8226 cells co-cultured with OCs, compared with the cells without OCs (Fig. 6A). In addition, increased c-Myc and reduced c-Jun in these cells were also consistent with the results obtained from the RPPA data.

To validate the different signal transduction of OCs-primed myeloma cells under normoxia and hypoxia conditions, we did western blot analysis for some key proteins shown in the inferred signaling pathway (Fig. 6B). The protein levels of phosphorylated-FAK, fibronectin, c-Myc and p53 were increased following treatment with hypoxia.

### Prediction of drug treatment effects based on the state transition analysis

A great advantage of discrete dynamic modeling is its ability to predict the outcomes of *system perturbations*. In dynamic Boolean network, the future state of each node is determined by the current states of its parental nodes via Boolean-operation-based transfer functions^36^. In this study, we considered three possible states for each protein in our model and the transfer functions were implemented by a set of integer linear constraints, which were generated according to the topology of inferred cell-specific pathway network (see Methods).

Our analysis above has shown the differential response of myeloma cells to oxygen tension in the presence or absence of OCs. Therefore, therapeutic approaches should also be taken into account of the complex heterogeneous microenvironment in the bone marrow. Considering the signaling molecules in our RPPA dataset were significantly changed at time point 24 h (Supplementary Fig. S4 and S5), we chose the predicted states of all the proteins at 24 h as an initial state of signaling network to study drug treatment effects (Figs 4 and 5B). The details about network state transition was described in Supplementary Text S5.

PI3K/AKT and Integrin/FAK-associated pathways were involved in regulation of myeloma cell proliferation, anti-apoptosis, cell cycle arrest, and migration under normoxic and hypoxic conditions, respectively (Figs 4 and 5). Therefore, we simulated the combined treatment with PI3K and integrin inhibitors on the inferred myeloma-specific pathway networks. The simulated drug effects are shown in Fig. 7. Figure 7A shows the simulated drug effect in normoxia. Comparing with the network shown in Fig. 4, PI3K/AKT, MEK/ERK, and JAK/STAT pathways were inhibited and NF-κB, S6, c-Myc, and c-Jun also down-regulated accordingly following the combined treatment, suggesting cell proliferation process was suppressed. The expression of pro-apoptotic factor BIM was up-regulated. These findings suggested that PI3k inhibition potentially reduced cell proliferation and enhanced apoptosis in the myeloma cells^37^,^38^. Figure 7B represents the simulated drug effect in hypoxia. The combined treatment with PI3k and integrin inhibitors led to a blockage of PKC and FAK pathways, potentially reducing migration and invasion of myeloma cells^39^. In the meantime, c-Myc was also down-regulated after treatment, suggesting an inhibition of cell growth. The state transition of signaling networks was solved by constraints (31–48) in Supplementary Text S5 and the fixed point (steady state) for both networks occurred after 5 and 2 steps, respectively. The expressions of all the proteins involved in above two networks were listed in Supplementary Table S2 and S3. Our drug simulation indicated that combination of PI3K and integrin inhibitors potentially reduced the myeloma cell proliferation in normoxia and inhibited cell migration in hypoxia. These predicted results were consistent with the previously reported results^40^,^41^.

## Discussion

In this paper, we presented a computational approach to infer the osteoclast-mediated myeloma cell-specific pathways in BM heterogeneous microenvironment and predict drug treatment effects on myeloma cells. Based on the differential expression of signaling proteins, we combined the enriched pathways from IPA with the related pathways described in the literatures to build up two generic pathway maps of myeloma cells. We then used our DILP approach for pathway topology optimization and eventually inferred the myeloma cells-specific pathway maps in normoxia and hypoxia. The cell-specific pathway maps were optimized by fitting generic pathway networks to the proteomics data collected experimentally. Each of our generic pathway maps was mainly built with the proteins which were differentially expressed under two conditions (for example, in the presence/absence of OCs), therefore, the cell-specific network inferred by DILP was used to clarify the significant differences of myeloma intracellular signaling response induced by different cellular contexts. A parts of signaling pathways in the inferred network might be represented as un-changed due to they both were up-regulated or down-regulated under both conditions.

An important aspect of the proposed approach is its practicability in inferring myeloma cell-specific pathways. Although ordinary differential equations (ODEs) are commonly used to model dynamics of signaling networks; estimating parameters using ODE models is quite challenging. Our approach, based on discrete modeling, has simplified the representation of signaling pathway topology.

For inference of the OC-mediated myeloma-specific pathways under the normoxic or hypoxic conditions, the optimization procedure of DILP approach contained about 2862 constraints and 1116 variables (81 integer variables and 1035 binary variables). About 673 constraints and 366 variables in each step of state transition were used for optimizing drug treatment effect. Compared to other systems modeling approaches (such as ODE-based method), our approach, based on linear constraints, can simplify the optimization process and quickly search an optimal solution in an allowable subspace. Even though this approach was scaled up to larger signaling networks, it still would be efficient because a linear programming problem can be solved in linear time when the dimension is fixed^42^.

When the proposed approach was applied on the RPPA data, fitting precision of the inferred cell-specific pathways was high (Supplementary Table S1). As shown in the Supplementary Table S1, the inferred cell-specific pathways were fitted well with the data obtained at 24 h post-treatment. In addition, several key factors were significantly altered at 24 h and no difference seen at 5 h or 48 h, indicating that the intracellular signals regulated by cell-cell interaction appeared to be time-dependent. The fitting curves in Supplementary Fig. S4-S5 have also shown the similar changes.

Furthermore, the DILP approach was also applied to study the drug treatment effects on myeloma cells based on the well-defined concept of state transition in dynamic Boolean network. Given an initial state of the specific pathways of myeloma cells, our DILP system was used to infer the future steady state (dynamic attractor) of the signaling network after the potential targets were inhibited by drugs. The simulation results of DILP approach indicated that treatment cells with the combined PI3K and integrin inhibitors potentially blocked the proliferation- and migration-associated signaling pathways and increase expression of apoptosis-associated proteins in myeloma cells. Early studies have shown that treatment myeloma cell with PI3K inhibitor led to a considerable induction of apoptosis, and inhibition of proliferation as well^33^,^34^. Moreover, integrin pathway is significantly up-regulated in MM, which plays a critical role in angiogenesis, migration, and invasion^43^. Puente *et al.* summarized that cell signaling targeted therapies (PI3K/AKT, p38, HDAC, and Wnt) and strategies targeting the tumor microenvironment (such as integrin, hypoxia, and angiogenesis) were currently two types of potential therapeutic strategies. The combination of these two therapeutic agents has improved outcomes for MM patients^41^. **SF1126**, an integrin-targeted PI3K inhibitor, has potent antitumor activity against multiple myeloma *in vitro* and *in vivo*^40^. Treatment with SF1126 on MM appeared to affect the tumor microenvironment by inhibiting angiogenesis^40^. In addition, some previous works reported that treatment of SF1126 on glioma and neuroblastoma cells blocked integrin-mediated migration^44^,^45^. Treatment of SF1126 on renal cell carcinoma results in marked inhibition of tumor growth via PI3K/AKT signaling pathway and profound inhibition of integrin-mediated migration^46^. Our prediction of drug effects is consistent with experimental results from others.

In summary, we constructed a computational framework using a systems biology approach to infer large-scale signaling pathways and predict dynamic behavior of the signal transduction system with time-series proteomics data.

## Methods

### Summary of the computational approach DILP

**Figure 1:**
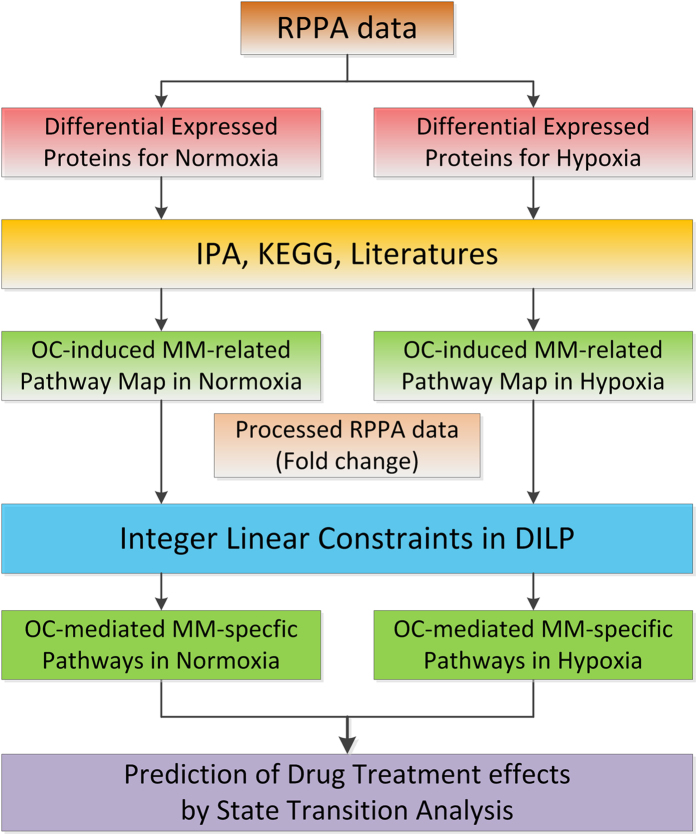
Flowchart of the proposed DILP approach.

**Figure 2:**
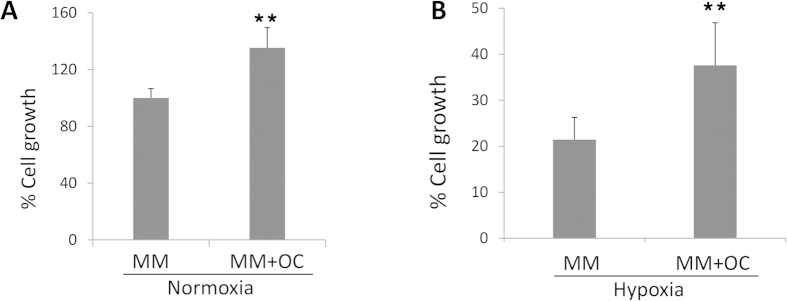
Effects of osteoclasts on myeloma cell. RPMI 8226 myeloma cells were cultured in the presence or absence of OCs under normoxia (21% O_2_, 5% CO_2_) and hypoxia (5% O_2_, 5% CO_2_) conditions. Myeloma cells were harvested at 72 h centrifugation. Myeloma cell growth was measured using dsDNA assay. Panel A shows myeloma growth in the presence or absence OCs under normoxic condition. Panel B shows myeloma cell growth in the presence or absence of OCs under hypoxic condition. ^**^means p < 0.01.

**Figure 3:**
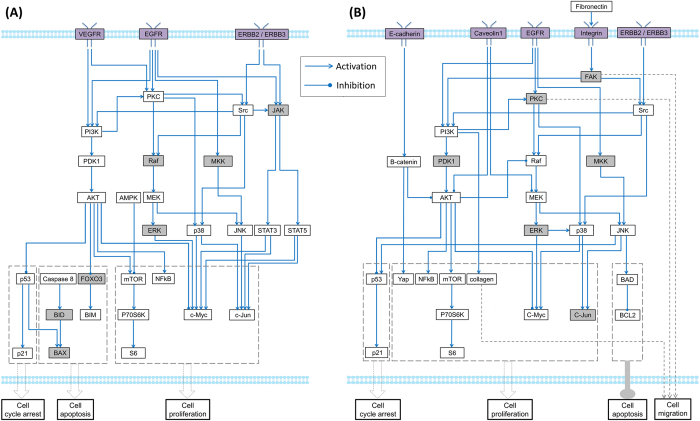
Generic pathway maps of myeloma cells in the presence of osteoclasts in normoxia and hypoxia. (**A**) OC-mediated myeloma cell-related generic pathway map in normoxia; (**B**) OC-mediated myeloma cell-related generic pathway map in hypoxia. The nodes with a dark color were predicted using our model.

**Figure 4:**
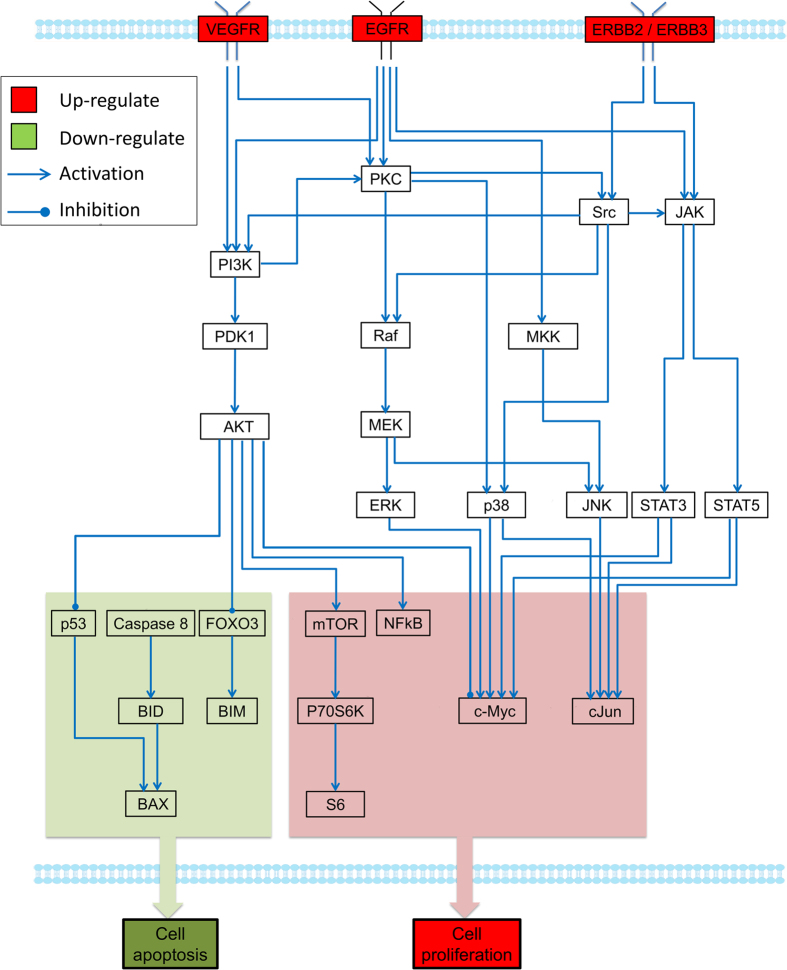
Inferred specific pathways of myeloma cells in the presence/absence of OCs under normoxic condition. Our model predicted that the functional module with red color was up-regulated, which potentially increases of cell proliferation and decrease of cell apoptosis. Similarity, the module with green color was down-regulated, which indicated the decrease of apoptosis. The predicted states of key proteins in this network were represented in detail in Supplementary Fig. S4.

**Figure 5:**
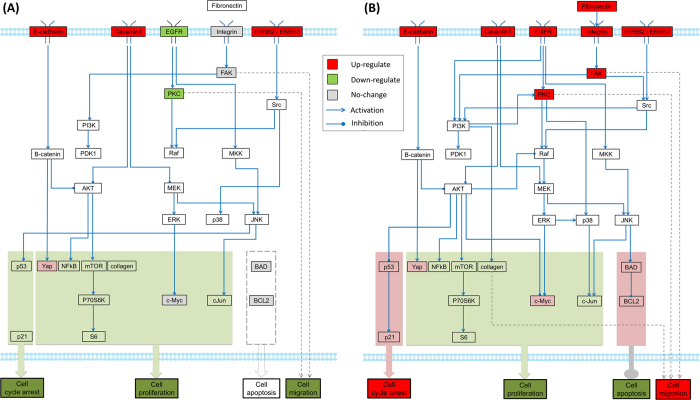
Inferred specific pathways of myeloma cells in the presence/absence of OCs under hypoxic condition. Several functional modules were highlighted to indicate the changes of phenotypes under different cellular contexts. (**A**) The specific pathways of myeloma cells in the absence of OCs under hypoxic condition. (**B**) The specific pathways of myeloma cells in the presence of OCs under hypoxic condition. The predicted states of key proteins in both networks were displayed in Supplementary Fig. S5 (A,B).

**Figure 6:**
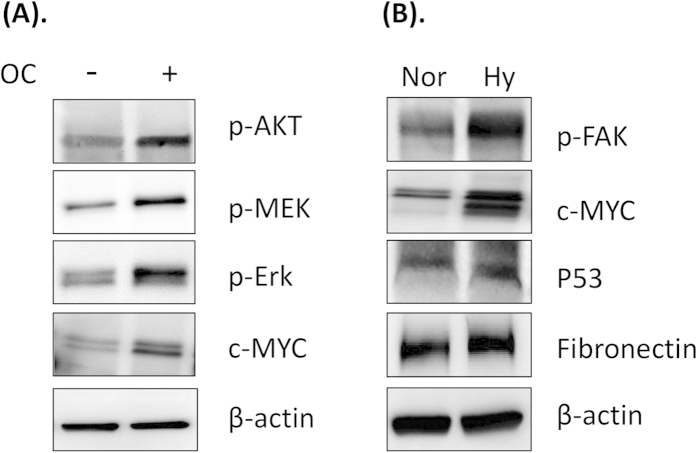
Experimental validation of key proteins involved in the inferred signaling pathways. (**A**) RPMI 8226 myeloma cells were cultured in the presence or absence of OCs under normoxic condition for 24 h. Cell lysates were collected and subjected to western blot analysis with p-Akt, p-MEK, p-ERK and c-MYC antibodies. (**B**) Myeloma cells were cultured in the presence OCs under normoxic and hypoxic conditions for 24 h. Myeloma cell lysates were subjected to western blot analysis with the FAK, c-MYC, p53 and fibronectin antibodies.

**Figure 7:**
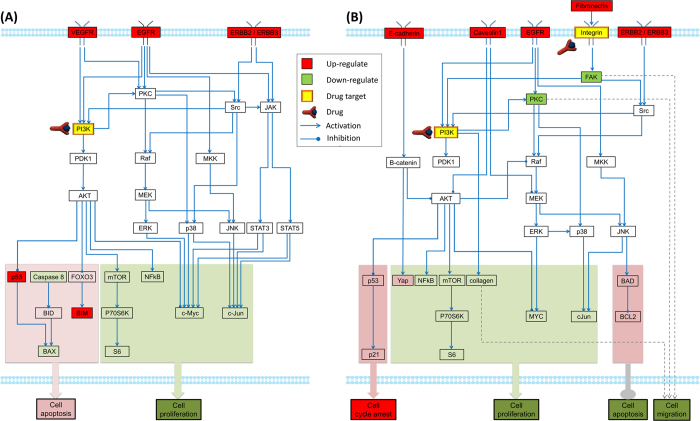
Simulation of treatment effects by perturbing the inferred cell-specific pathways with combination of PI3K and integrin inhibitors. The changes of downstream modules were highlighted. (**A**) Predicted treatment effects of PI3K and integrin inhibitors on OC-mediated myeloma cells-specific pathways in normoxia; (**B**) Predicted treatment effects of PI3K and integrin inhibitors on OC-mediated myeloma cells-specific pathways in hypoxia.

In this study, we designed DILP approach to model myeloma cell growth and survival, and simulate perturbation effects of drugs in the presence/absence of OCs and in the normoxic/hypoxic microenvironment (Fig. 1). The modeling approach consisted of three major steps, including construction of generic pathway maps, inference of cell-specific pathways by DILP; and prediction of drug treatment effects by state transition analysis.

#### Step 1: Construction of generic pathway maps

Firstly, differentially expressed proteins were identified from the experimental proteomics data. We then selected the enriched signaling pathways from the canonical pathway database through the use of Ingenuity Pathway Analysis (IPA) based on the differentially expressed proteins. Secondly, the generic signaling pathway maps were manually built by merging the enriched signaling pathways with myeloma cell-related pathways reported in the literatures.

**Figure 8:**
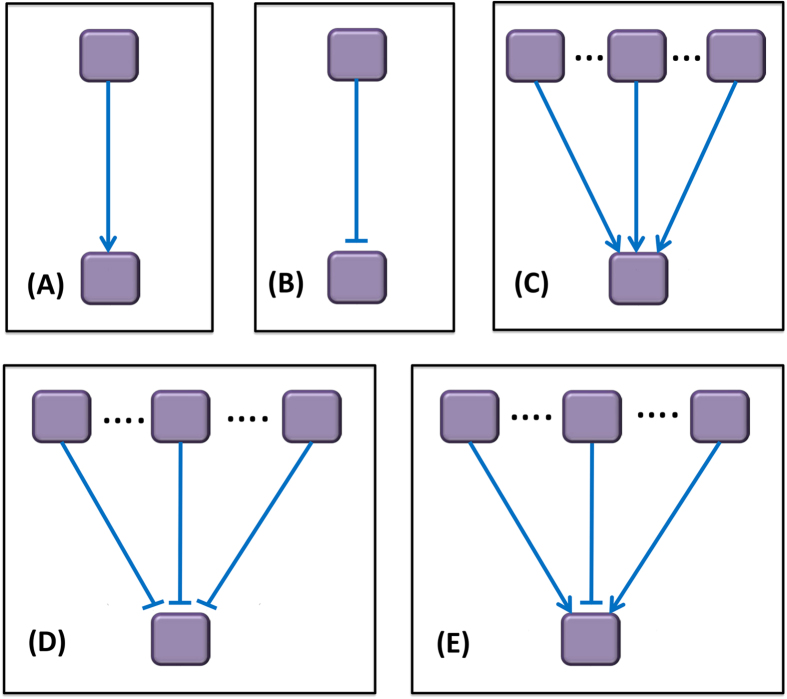
Five cases of linking patterns of signaling proteins in pathway network topology. (**A**) single activation; (**B**) single inhibition; (**C**) multiple activations; (**D**) multiple inhibition; (**E**) mixed reactions. (**A**,**B**) show that, the state of downstream protein is determined by its unique parental node through single reaction of activation/inhibition, respectively; In (**C**,**D**) the state of a downstream protein can be changed if at least one of the upstream proteins is up-regulated/down-regulated and others are un-changed. In Fig. 8E, the state of downstream protein might be un-changed (0), up-regulated (1) or down-regulated (−1) if it has the potential of being both up- and down-regulated by its parental nodes, simultaneously.

The signaling pathway maps were presented as a discrete network, consisting of a set of nodes and directed edges. Nodes in the pathway networks represented signaling proteins with their discrete values 1,−1, and 0, which standed for “up-regulation”, “down-regulation”, and “no-change”, respectively. The edges indicate signal reactions. There were two types of reactions: activation (“→”) and inhibition (“
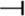
”), which were encoded by integer variables (1 or −1). In this study, we summarized five cases of linking patterns of signaling proteins which were common in most of pathway network topologies (Fig. 8). Figure 8 indicated that the state of a downstream protein was determined by a set of upstream proteins and the linking pattern between them. Figure 8A,B indicates that, the downstream protein is regulated by its unique parental node through a single reaction (activation or inhibition), respectively; In Fig. 8C,D, the states of a downstream protein could be modified if at least one of the parental proteins was up-regulated/down-regulated. In Fig, 8E, a downstream protein was up-regulated (1), down-regulated (−1) or unchanged (0), if it had been both up- and down-regulated by activation and inhibition, simultaneously. In our model, the states of all the nodes and connected edges in the pathway network meets the ***state consistent rules*** (see Supplementary Text S1).

#### Step 2: Inference of cell-specific pathways by DILP

A signaling pathway network is defined as a set of signaling proteins *P* = {1, 2, …,  *j*, …, *n*_*s*_} and reactions *E* = {1, 2, …, *i*, …, *n*_*r*_}. All of the proteins were measured at several time points, indexed by the set *T* = {*t*_1_, *t*_2_, …, *t*_*L*_}. A discrete variable *x*_*j,k*_ ∈ {−1, 0, 0} indicates whether the protein *j* (*j *∈ *P*) is up-regulated (*x*_*j,k*_ = 1), down-regulated (*x*_*j,k*_ = −1), or un-changed (*x*_*j,k*_ = 0) at the time point *k*, in which *k *∈ *T*. The reaction *i* (*i ∈ E*) can be represented as *u* → *d* (activation) or *u* 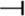 *d*(inhibition), where *u* and *d* are the upstream and downstream proteins of this reaction, respectively (*u*, *d* ∈ *P*). The *impact* (“positive regulatory” or “negative regulatory”) of the upstream protein *u* on downstream protein *d* is described as the regulating effect from *u* to *d* when protein *u* is up- or down-regulated. They will be used to design the constraints of the reactions in the inferred signaling pathways (Supplementary Text S2).

To find an optimal set of reactions from the original generic pathway map, we have introduced the binary variable *y*_*i*_ and *z*_*i,k*_. The variable *y*_*i*_ denotes 1 if the reaction *i* is removed in the inferred cell-specific pathway network, and 0 else wise. The variable *z*_*i,k*_ denotes 0 if the reaction *i* (*i *∈* E*) takes place at the time point *k*, and 1 else wise. The state of *z*_*i,k*_ may affect the fitting error between experimentally measured and predicted values of proteins. Moreover, formulas (1–2) reflect how the state of reaction *i* at time point *k* (*z*_*i,k*_) constrains the presence of this reaction in the inferred cell-specific pathway network. Formula (1) indicates that reaction *i* is present in the cell-specific pathway network if it takes place at least at one time point. Formula (2) denotes that the reaction *i* is not included in the cell-specific pathway network if this reaction doesn’t occur at all of the time points.


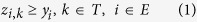



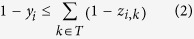


To infer cell-specific pathway networks, we developed a time-series-data-driven Integer Linear Programming (simply called as dynamic ILP or DILP) approach to minimize the differences between experimentally measured and predicted values of signaling proteins, as well as to obtain a minimized sub-network of original generic pathway map. The objective function is defined as:


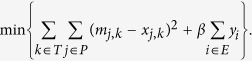


In this objective function, the first term represents the fitting error between experimentally measured and predicted values and the second term denotes a set of reactions from the original generic signaling network. The measured and predicted values of *j*–th protein at time point *k* were denoted as *m*_*j,k*_,*x*_*j,k*_ ∈ {−1, 0, 1}, respectively. When *m*_*j,k*_ and *x*_*j,k*_ are equal, the value of term (*m*_*j,k*_−*x*_*j,k*_)^2^ will be 0; otherwise it is either 1 or 4. Hence, optimization of the above objective function might induce local-optimal solution because of the non-uniform distribution of the term (*m*_*j,k*_−*x*_*j,k*_)^2^. In order to address this bias, binary variable *a*_*j,k*_ (taking value of 0 or 1) was designed as the difference between *m*_*j,k*_ and *x*_*j,k*_ as following: *a*_*j,k*_ will be 1 if *m*_*j,k*_ is not equal to *x*_*j,k*_, and 0 else wise. Then the term (*m*_*j,k*_−*x*_*j,k*_)^2^ in above objective function was replaced by *a*_*j,k*_. The calculation of *a*_*j,k*_ is implemented by constraints (26-27) as shown in Supplementary Text S2. Finally, the above objective function can be simplified as formula (3).


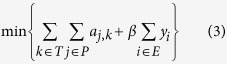


The negative constant *β* in formula (3) is used to obtain a minimum sub-graph of the generic pathway maps as the finalized cell-specific pathways (here, we have 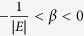), in which 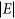 is the number of reactions in the network. In the optimization procedure, the predicted states of proteins and reactions in signaling pathway network meet ***State consistent rules***(see Supplementary Text S1). All these rules were implemented using our developed constraints which were presented in Supplementary Text S2.

In addition, minimizing the optimal network topology by edge removals might eliminate some reactions, leading to some phosphor-signals not being transduced into downstream proteins (such as two examples shown in Supplementary Fig. S6-S7). After obtaining a single minimized sub-graph of generic pathway network via *DILP* approach, we designed a strategy for searching the missing edges. The missing edges were added to the optimal network obtained from formula (3) if the *goodness of fit* was un-changed. The details of addition of missing edges were described in Supplementary Text S4.

The DILP formulations presented above were implemented in our Matlab-based software tool *DILPMAT (Http://ctsb.is.wfubmc.edu/publications/DILPMAT)*. This toolbox was developed basing on GUROBI optimizer^31^, which is a well-known Mathematical Programming Solver. The optimization of formula (3) using *DILPMAT* will deliver an optimal sub-network (cell-specific pathways) of the generic pathways which can best explain the experimental time series proteomics data.

#### Step 3: Prediction of drug treatment effects by state transition analysis

Based on the established network topological structure and transfer functions (Boolean operations), state transition analysis in *Boolean networks* is a kind of approach to predict the future state of each node from the current states of its parental nodes^36^,^47^,^48^. The assumption of state transition is that: the state of protein *j* at time point *k* + 1 (*x*_*j,k*+1_) is associated with the states of its parental proteins at time point *k*^48^,^49^. Thus we predicted the state of pathway network at time point *k* + 1 from the state at time point *k* through state transition, following perturbation of the cell-specific pathway network with drugs (formula (4)). Given the inferred cell-specific pathway network topology *G* and the states of all the proteins involved in the network at time point *k*: *X*_*k*_ = [*x*_1,*k*_, *x*_2,*k*_, …, *x*_*n,k*_] (n is the total number of proteins in the network), we can obtain the states of these proteins at time point *k* + 1 using the following formula:


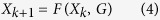


where *k* = 1, 2, …, *L*; *x*_*j,k*_∈{−1, 0, 1}; and *F* is a set of transfer functions to change the signaling network from one state to another. In Boolean networks, transfer functions are denoted by using logical expressions via Boolean operators^48^,^50^. However, it is difficult to represent the transfer functions using an established mathematic expression if each signaling protein has three possible states (1, −1, or 0)^17^. In our study, the transfer functions *F* were represented by a set of integer linear constraints. The details of these constraints involved in the transfer functions *F* were described in Supplementary Text S5.

Let’s use *X*_0_ = {*x*_1, 0_, *x*_2,0_, …, *x*_*j*,0, …,_ *x*_*n*,0_} (*x*_*j,*0_ ∈ {−1, 0, 1}) to denote a measured state of signaling network without any intervention or treatment. When cells are treated by an inhibitor, we assume protein *j* is targeted by this inhibitor, and then the state of above signaling network after treatment is changed as *X*_0_ = {*x*_1,0_, *x*_2,0_, …, −1_, …,_ *x*_*n*,0_}. We used *X*_0_ as the initial state to predict the performance of the inhibition or perturbation. We then used formula (4) to calculate the next state (*X*_1_) of *X*_0_ and repeat this process until the signaling network reached to steady state^50^. We eventually generated a set of states {*X*_1_, *X*_2_, …, *X*_*A*_}. *X*_*A*_ is regarded as the final effects of drug treatment in cell-specific pathway network.

### Cell culture and analysis

#### Cell culture

Human myeloma cell line RPMI-8226 was obtained from the American Type Culture Collection (Rockville, MD, USA). The cells were routinely maintained in RPMI 1640 medium (DMEM) containing 10% heat-inactive bovine calf serum, 2 mM L-glutamine, 100 IU/ml penicillin and 100 *μ*g/ml streptomycin (all from Invitrogen, Gaithersburg, MD, USA) at 37 °C with 5% CO_2_ in air.

#### Isolation of peripheral blood mononuclear cells (PBMCs)

PBMCs were isolated from buffy coat (Gulf Coast Blood Center, Houston, TX) using Ficoll-Paque density gradient centrifugation. Briefly, whole blood was mixed with an equal amount of 1 X PBS (Bio-Rad, Hercules, CA), and layered over Ficoll-Paque. The PBMC layer was removed, washed, and centrifuged twice with Hank’s Balanced Salt Solution (HBSS) (Sigma-Aldrich, St. Louis, MO). Totally 20 buffy coat samples were used in this study.

#### Generation of human osteocalst-like multinucleated cells (OCs)

OCs were generated *in vitro* according to the previously described procedures^51^,^52^,^53^. PBMCs were seeded in 24 well Osteo Assay surface plates (Corning, Tewksbury, MA) for 3 h in alpha-MEM medium (Life Technologies, Gaithersburg, MD) supplemented with 10% fetal bovine serum. Non-adherent cells were discarded by rinsing with 1 X PBS and the remaining adherent cells were cultured in a complete alpha-MEM medium containing 10% FBS, 50 ng/ml M-CSF and 30 ng/ml recombinant RANKL at 37 °C in a humidified atmosphere of 21%O_2_ and 5% CO_2_ for 2–3 weeks. The generation of OCs was determined by staining the cells with tartrate-resistant acid phosphatase (TRAP). The TRAP was performed using a commercial acid phosphatase leucocyte kit (Sigma, St Louis, MO). Images were taken using an Olympus IX83 microscope.

#### Coculture of myeloma cells and osteoclasts

For co-culture experiments, primary OCs cultured in a 24-well plate were washed twice with 1 X PBS to remove non-adherent cells. RMPI-8266 cells suspended in osteoclast culture medium were added to the OC culture plate (2 × 10^5^ cells/per well) and incubated in normoxic (21% O_2_) and hypoxic (5% O_2_) conditions. For the hypoxic culture, medium was pre-incubated in a hypoxic incubator for 2 h before use. We used a tri-gas incubator (Thermo Scientific Heracell™ 150i CO_2_ incubator) and nitrogen gas was employed for the regulation of O_2_ concentration. RMPI-8266 cells were rinsed using PBS and collected by centrifugation for 10 min at 300 g at expected time points.

#### dsDNA quantitation

Double strand DNA (dsDNA) was used for estimation of cell numbers and quantified using Quant-iT PicGreen assay kit (Invitrogen). Briefly, at day 3 following treatment, cells were collected by centrifugation. After washing with 1 X PBS for three times, 500 μl of 0.05% Triton-X in PBS was added to lyse cells. Cell lysates were stored at −80 °C until further analysis. Each thawed sample was sonicated for 5 s using an ultrasonic cell disrupter (Fisher Scientific, pittsburgh, PA). Fifty μl of the cell lysates were mixed with an equal volume of PicoGreen working solution and incubated in the dark for 5 min. The plate was then read on a SpectraMax M2 fluorescence microplate reader (Molecular Devices Inc., Sunnyvale, CA) at excitation and emission wavelengths of 485 and 535 nm, respectively. The dsDNA contents were calculated according to a standard curve generated using a set of double stranded DNA standard.

#### Reverse Phase Protein Array (RPPA)

RPRA was performed by RPPA Core Facility of MD Anderson Cancer Center (Houston, TX) as described previously^19^. We had two replicates for each samples and there was no significant difference observed between two replicates. RPMI-8266 cells were harvested at 5 h, 24 h and 48 h after treatments. Cells were lysed with a RIPA buffer (150 mM NaCl, 1% NP-40, 1% sodium deoxycolate in 50 mM Tris-HCl, pH 7.5). The cell lysates were centrifuged at 14,000 rpm for 10 min at 4 °C, supernatants collected and protein concentration determined by Bradford method (Biorad, Hercules, CA). The cell lysates were then mixed with SDS sample buffer (250 mmol/l Tris, pH 7.4, 2% w/v SDS, 25% v/v glycerol and 10% v/v 2-mercaptoethanol) and boiled for 5 min. The samples were then sent to RPPA core facility in MD Anderson Cancer Center for analysis. RPPA assay includes 172 antibodies which recognized proteins associated with cell surface growth factors and receptors, common signaling pathway molecules, steroid hormone receptors, and proliferation and apoptosis. The antibodies used for RPPA analysis are listed on the MD Anderson website (http://www.mdanderson.org/).

#### Western blot

The cell lysates collected above were mixed with an equal volume of SDS sample buffer, heated to 95 °C for 5 minutes, and chilled at 4 °C for 10 minutes. 30 μg proteins were loaded on an 4–15% SDS-polyacrylamide gel (Bio-Rad), run for around 90 minutes at 100 v, and then transferred to a nitrocellulose membrane (GE Healthcare) in a transfer buffer containing 25 mmol/l Tris, pH 8.8, 192 mmol/l glycine, and 10% v/v methanol (60 min at 100 v). All washing, blocking and antibody solutions were prepared in TBST (the details of antibodies see Supplementary Table S4). Membranes were blocked in 5% BSA (bovine serum albumin) for 1 h, followed by overnight incubation with primary antibodies. Membranes were then washed three times, followed by secondary antibody incubation for 1 h in 5% BSA. After washing 3 times with TBST, the blots were probed using an enhanced chemiluminescence system (Cell signaling) and imaged on a ChemQ imager. Densitometrical analysis was performed following acquisition using Image J software (NIH).

## Additional Information

**How to cite this article**: Ji, Z. *et al.* Systemic modeling myeloma-osteoclast interactions under normoxic/hypoxic condition using a novel computational approach. *Sci. Rep.* **5**, 13291; doi: 10.1038/srep13291 (2015).

## Supplementary Material

Supplementary Information

## References

[b1] Hu J., Van Valckenborgh E., Menu E., De Bruyne E. & Vanderkerken K. Understanding the hypoxic niche of multiple myeloma: therapeutic implications and contributions of mouse models. Dis. Model Mech. 5, 763–771 (2012). 23115205 10.1242/dmm.008961 PMC3484859

[b2] Manier S., Sacco A., Leleu X., Ghobrial I. M. & Roccaro A. M. Bone marrow microenvironment in multiple myeloma progression. J. Biomed Biotechnol. 2012, 157496 (2012). 23093834 10.1155/2012/157496 PMC3471001

[b3] Hu J. *et al.* Targeting the multiple myeloma hypoxic niche with TH-302, a hypoxia-activated prodrug. Blood 116, 1524–1527 (2010). 20530289 10.1182/blood-2010-02-269126

[b4] Azab A. K. *et al.* Hypoxia promotes dissemination of multiple myeloma through acquisition of epithelial to mesenchymal transition-like features. Blood 119, 5782–5794 (2012). 22394600 10.1182/blood-2011-09-380410 PMC3382938

[b5] Ribatti D., Moschetta M. & Vacca A. Microenvironment and multiple myeloma spread. Thrombosis Research 133, S102–S106 (2014). 24862128 10.1016/S0049-3848(14)50017-5

[b6] Danet G. H., Pan Y., Luongo J. L., Bonnet D. A. & Simon M. C. Expansion of human SCID-repopulating cells under hypoxic conditions. J. Clin. Invest. 112, 126–135 (2003). 12840067 10.1172/JCI17669 PMC162287

[b7] Parmar K., Mauch P., Vergilio J. A., Sackstein R. & Down J. D. Distribution of hematopoietic stem cells in the bone marrow according to regional hypoxia. Proc. Natl. Acad. Sci. USA. 104, 5431–5436 (2007). 17374716 10.1073/pnas.0701152104 PMC1838452

[b8] Levesque J. P. *et al.* Hematopoietic progenitor cell mobilization results in hypoxia with increased hypoxia-inducible transcription factor-1 alpha and vascular endothelial growth factor A in bone marrow. Stem Cells 25, 1954–1965 (2007). 17478585 10.1634/stemcells.2006-0688

[b9] Zhao X. *et al.* Hypoxia-inducible factor-1 promotes pancreatic ductal adenocarcinoma invasion and metastasis by activating transcription of the actin-bundling protein fascin. Cancer Res. 74, 2455–2464 (2014). 24599125 10.1158/0008-5472.CAN-13-3009

[b10] Zhou J., Schmid T., Schnitzer S. & Brune B. Tumor hypoxia and cancer progression. Cancer Lett. 237, 10–21 (2006). 16002209 10.1016/j.canlet.2005.05.028

[b11] Li B. *et al.* Effect of hypoxia on the growth and apoptosis of the human multiple myeloma cell lines RPMI8226. Zhonghua Xue Ye Xue Za Zhi 35, 664–666 (2014). 25052617 10.3760/cma.j.issn.0253-2727.2014.07.023

[b12] Li B. Z., Zhuang W. Z., Chen P. & Fu J. X. Inhibition of hypoxia-inducible factor-1 alpha in RPMI8226 myeloma cells results in reduced tumor growth in nude mice. Zhonghua Xue Ye Xue Za Zhi 29, 247–251 (2008). 18843979

[b13] Peng H., Wen J., Li H., Chang J. & Zhou X. Drug inhibition profile prediction for NFkappaB pathway in multiple myeloma. PLoS One 6, e14750 (2011). 21408099 10.1371/journal.pone.0014750 PMC3051063

[b14] Aldridge B. B., Burke J. M., Lauffenburger D. A. & Sorger P. K. Physicochemical modelling of cell signalling pathways. Nat. Cell Biol. 8, 1195–1203 (2006). 17060902 10.1038/ncb1497

[b15] Samaga R., Saez-Rodriguez J., Alexopoulos L. G., Sorger P. K. & Klamt S. The logic of EGFR/ErbB signaling: theoretical properties and analysis of high-throughput data. PLoS Comput. Biol. 5, e1000438 (2009). 19662154 10.1371/journal.pcbi.1000438 PMC2710522

[b16] Mitsos A. *et al.* Identifying Drug Effects via Pathway Alterations using an Integer Linear Programming Optimization Formulation on Phosphoproteomic Data. PLoS Comput. Biol. 5, e1000591 (2009). 19997482 10.1371/journal.pcbi.1000591 PMC2776985

[b17] Melas I. N., Samaga R., Alexopoulos L. G. & Klamt S. Detecting and Removing Inconsistencies between Experimental Data and Signaling Network Topologies Using Integer Linear Programming on Interaction Graphs. Plos Comput. Biol. 9, e1003204 (2013). 24039561 10.1371/journal.pcbi.1003204 PMC3764019

[b18] Ji Z. *et al.* Integrating genomics and proteomics data to predict drug effects using binary linear programming. PLoS One 9, e102798 (2014). 25036040 10.1371/journal.pone.0102798 PMC4103865

[b19] Peng H. *et al.* Characterization of p38 MAPK isoforms for drug resistance study using systems biology approach. Bioinformatics 30, 1899–1907 (2014). 24618474 10.1093/bioinformatics/btu133 PMC4071201

[b20] Colla S. *et al.* Low bone marrow oxygen tension and hypoxia-inducible factor-1alpha overexpression characterize patients with multiple myeloma: role on the transcriptional and proangiogenic profiles of CD138(+) cells. Leukemia 24, 1967–70 (2010). 20811474 10.1038/leu.2010.193

[b21] Cicione C. *et al.* Effects of severe hypoxia on bone marrow mesenchymal stem cells differentiation potential. Stem Cells Int 2013, 232896 (2013). 24082888 10.1155/2013/232896 PMC3777136

[b22] Koller M. R., Bender J. G., Miller W. M. & Papoutsakis E. T. Reduced oxygen tension increases hematopoiesis in long-term culture of human stem and progenitor cells from cord blood and bone marrow. Exp Hematol 20, 264–70 (1992). 1544397

[b23] Boregowda S. V. *et al.* Atmospheric oxygen inhibits growth and differentiation of marrow-derived mouse mesenchymal stem cells via a p53-dependent mechanism: implications for long-term culture expansion. Stem Cells 30, 975–87 (2012). 22367737 10.1002/stem.1069 PMC3683654

[b24] Hideshima T. & Anderson K. C. Molecular mechanisms of novel therapeutic approaches for multiple myeloma. Nature Reviews Cancer 2, 927–937 (2002). 12459731 10.1038/nrc952

[b25] Saha M. N. *et al.* Targeting p53 via JNK pathway: a novel role of RITA for apoptotic signaling in multiple myeloma. PLoS One 7, e30215 (2012). 22276160 10.1371/journal.pone.0030215 PMC3262803

[b26] Demchenko Y. N. & Kuehl W. M. A critical role for the NFkB pathway in multiple myeloma. Oncotarget 1, 59–68 (2010). 20890394 10.18632/oncotarget.109 PMC2947827

[b27] Pene F. *et al.* Role of the phosphatidylinositol 3-kinase/Akt and mTOR/P70S6-kinase pathways in the proliferation and apoptosis in multiple myeloma. Oncogene 21, 6587–6597 (2002). 12242656 10.1038/sj.onc.1205923

[b28] Gandarillas A. & Watt F. M. c-Myc promotes differentiation of human epidermal stem cells. Genes Dev. 11, 2869–2882 (1997). 9353256 10.1101/gad.11.21.2869 PMC316650

[b29] Mitsiades C. S. *et al.* TRAIL/Apo2L ligand selectively induces apoptosis and overcomes drug resistance in multiple myeloma: therapeutic applications. Blood 98, 795–804 (2001). 11468181 10.1182/blood.v98.3.795

[b30] Mitsiades N. *et al.* Apoptotic signaling induced by immunomodulatory thalidomide analogs in human multiple myeloma cells: therapeutic implications. Blood 99, 4525–4530 (2002). 12036884 10.1182/blood.v99.12.4525

[b31] Werbos L., Kozma R., Silva-Lugo R., Pazienza G. E. & Werbos P. J. Metamodeling and the Critic-based approach to multi-level optimization. Neural Networks 32, 179–185 (2012). 22386785 10.1016/j.neunet.2012.02.036

[b32] Han S. *et al.* Activated hepatic stellate cells promote hepatocellular carcinoma cell migration and invasion via the activation of FAK-MMP9 signaling. Oncol. Rep. 31, 641–648 (2014). 24284889 10.3892/or.2013.2872

[b33] Kim N. G., Koh E., Chen X. & Gumbiner B. M. E-cadherin mediates contact inhibition of proliferation through Hippo signaling-pathway components. Proc. Natl. Acad. Sci. USA. 108, 11930–11935 (2011). 21730131 10.1073/pnas.1103345108 PMC3141988

[b34] Lau M. T., Klausen C. & Leung P. C. E-cadherin inhibits tumor cell growth by suppressing PI3K/Akt signaling via beta-catenin-Egr1-mediated PTEN expression. Oncogene 30, 2753–2766 (2011). 21297666 10.1038/onc.2011.6

[b35] Ljungman M. & Lane D. P. Transcription - guarding the genome by sensing DNA damage. Nat. Rev. Cancer 4, 727–737 (2004). 15343279 10.1038/nrc1435

[b36] Saadatpour A. & Albert R. Discrete dynamic modeling of signal transduction networks. Methods Mol. Biol. 880, 255–272 (2012). 23361989 10.1007/978-1-61779-833-7_12

[b37] Glauer J. *et al.* A novel selective small-molecule PI3K inhibitor is effective against human multiple myeloma *in vitro* and *in vivo*. Blood Cancer J. 3, e141 (2013). 24013662 10.1038/bcj.2013.37 PMC3789203

[b38] Ikeda H. *et al.* PI3K/p110 delta is a novel therapeutic target in multiple myeloma. Blood 116, 1460–1468 (2010). 20505158 10.1182/blood-2009-06-222943 PMC2938837

[b39] Wang X., Zhang Z. & Yao C. Targeting integrin-linked kinase increases apoptosis and decreases invasion of myeloma cell lines and inhibits IL-6 and VEGF secretion from BMSCs. Med Oncol 28, 1596–600 (2011). 20625942 10.1007/s12032-010-9616-y

[b40] De P. *et al.* An integrin-targeted, pan-isoform, phosphoinositide-3 kinase inhibitor, SF1126, has activity against multiple myeloma *in vivo*. Cancer Chemother Pharmacol 71, 867–81 (2013). 23355037 10.1007/s00280-013-2078-0 PMC3832139

[b41] de la Puente P., Muz B., Azab F., Luderer M. & Azab A. K. Molecularly targeted therapies in multiple myeloma. Leuk Res Treatment 2014, 976567 (2014). 24829804 10.1155/2014/976567 PMC4009206

[b42] Megiddo N. Linear-Programming in Linear Time When the Dimension Is Fixed. Journal of the Acm. 31, 114–127 (1984).

[b43] Podar K. & Anderson K. C. The pathophysiologic role of VEGF in hematologic malignancies: therapeutic implications. Blood 105, 1383–95 (2005). 15471951 10.1182/blood-2004-07-2909

[b44] Singh A. R., Joshi S., George E. & Durden D. L. Anti-tumor effect of a novel PI3-kinase inhibitor, SF1126, in (12) V-Ha-Ras transgenic mouse glioma model. Cancer Cell Int 14, 105 (2014). 25425962 10.1186/s12935-014-0105-9 PMC4243316

[b45] Peirce S. K. *et al.* The PI-3 kinase-Akt-MDM2-survivin signaling axis in high-risk neuroblastoma: a target for PI-3 kinase inhibitor intervention. Cancer Chemother Pharmacol 68, 325–35 (2011). 20972874 10.1007/s00280-010-1486-7 PMC3143317

[b46] Joshi S., Singh A. R. & Durden D. L. Pan-PI-3 kinase inhibitor SF1126 shows antitumor and antiangiogenic activity in renal cell carcinoma. Cancer Chemother Pharmacol 75, 595–608 (2015). 25578041 10.1007/s00280-014-2639-x

[b47] Cheng D. Z. & Qi H. S. State-Space Analysis of Boolean Networks. Ieee Transactions on Neural Networks 21, 584–594 (2010). 20172826 10.1109/TNN.2009.2039802

[b48] Chaves M., Albert R. & Sontag E. D. Robustness and fragility of Boolean models for genetic regulatory networks. J. Theor. Biol. 235, 431–449 (2005). 15882705 10.1016/j.jtbi.2005.01.023

[b49] Zhou X. *et al.* A Bayesian connectivity-based approach to constructing probabilistic gene regulatory networks. Bioinformatics 20, 2918–2127 (2004). 15145802 10.1093/bioinformatics/bth318

[b50] Albert R. & Wang R. S. Discrete dynamic modeling of cellular signaling networks. Methods Enzymol. 467, 281–306 (2009). 19897097 10.1016/S0076-6879(09)67011-7

[b51] Suzuki Y. *et al.* Osteoclast-like cells in an *in vitro* model of bone destruction by rheumatoid synovium. Rheumatology (Oxford) 40, 673–682 (2001). 11426026 10.1093/rheumatology/40.6.673

[b52] Abe M. *et al.* Osteoclasts enhance myeloma cell growth and survival via cell-cell contact: a vicious cycle between bone destruction and myeloma expansion. Blood 104, 2484–91 (2004). 15187021 10.1182/blood-2003-11-3839

[b53] Tanaka Y. *et al.* Myeloma cell-osteoclast interaction enhances angiogenesis together with bone resorption: a role for vascular endothelial cell growth factor and osteopontin. Clin Cancer Res 13, 816–23 (2007). 17289872 10.1158/1078-0432.CCR-06-2258

