# Supplementary material for: Systemic modeling myeloma-osteoclast interactions under normoxic/hypoxic condition using a novel computational approach
Source: Sci Rep. 2015 Aug 18;5:13291. doi: 10.1038/srep13291 (PMC4539608; doi:10.1038/srep13291)
Supplement: Supplementary Information [file srep13291-s1.pdf]

Title page of supplementary files

Systemic modeling myeloma-osteoclast interactions under normoxic/hypoxic condition using a novel computational approach

Zhiwei Ji[1,2 +], Dan Wu[2 +], Weiling Zhao[2], Huiming Peng[2], Deshuang Huang[1,*], Xiaobo Zhou[2,*]

[1] School of Electronics and Information Engineering, Tongji University, Shanghai, P.R. China 201804

[2] Division of Radiologic Sciences – Center for Bioinformatics and Systems Biology, Wake Forest School of Medicine, Medical Center Boulevard, Winston-Salem, NC, USA 27157

* Corresponding author: X.Z. (xizhou@wakehealth.edu), D.H. (dshuang@tongji.edu.cn)

+ The authors contributed equally to this work.

Supplementary files list:

Text S1 State consistent rules.

Text S2 Computational procedure: Inference of cell-specific pathways with DILP.

Text S3 Computational procedure: fitting precision of data (goodness of fit).

Text S4 Computational procedure: addition of missing edges into a single optimal solution.

Text S5 Computational procedure: prediction of drug treatment effects by state transition analysis.

Fig. S1 TRAP staining of OCs (20× amplification).

Fig. S2 HIF1 expression in myeloma cells under hypoxic condition.

Fig. S3 The heatmap of all the RPPA proteins in both conditions.

Fig. S4 The predicted states and measured states of some key factors in the inferred specific pathways of MM cells in normoxic condition.

Fig. S5 The predicted states and measured states of key factors in the inferred specific pathways of MM cells in hypoxic condition with the presence versus absence of OC.

Fig. S6 An example about the addition of missing edges.

Fig. S7 Another example about the addition of missing edges.

Table S1 The fitting precision of the proposed DILP model on the processed RPPA data.

Table S2 The expressions of all the proteins involved in the OC-mediated MM-specific pathways in normoxia (with the presence of OC) before and after treatment with PI3K inhibitor.

Table S3 The expressions of all the proteins involved in the OC-mediated MM-specific pathways in hypoxia (with the presence of OC) before and after treatment with combination of PI3K and integrin inhibitors.

Table S4 The details of the antibodies.

Supplementary Text

S1. State consistent rules

In our study, signaling pathway network was represented as a set of signaling proteins $P = \{1,2,\ldots,j,\ldots n_s\}$ and reactions $E = \{1,2,\ldots,i,\ldots n_r\}$. All of the proteins were measured at several time points, indexed by set $T = \{t_1, t_2, \ldots, t_L\}$. A discrete variable $x_{j,k} \epsilon \{-1,0,1\}$ indicates whether the protein j ($j \in P$) is up-regulated ($x_{j,k} = 1$), down-regulated ($x_{j,k} = -1$), or un-changed ($x_{j,k} = 0$) at time point k, in which $k \in T$. The reaction i ($i \in E$) can be represented as $u \rightarrow d$ (activation) or $u \dashv d$ (inhibition), where u and d are the upstream and downstream proteins of this reaction, respectively ($u, d \in P$). The states of proteins and reactions involved in a complicated signaling network should meet the state consistent rules. Here, we discussed five cases of linking patterns of signaling proteins which were common in most of pathway network topologies to demonstrate the details of state consistent rules (also see **Figure 8**).

Case 1: An activation edge indicates positive regulatory (**Figure 8A**).

a) If $x_d = -1$: there is a predecessor node u and an edge $u \rightarrow d$ with $x_u = -1$;

b) If $x_d = 1$: there is a predecessor node u and an edge $u \rightarrow d$ with $x_u = 1$;

c) If $x_d = 0$: node u has no predecessor; or an edge $u \rightarrow d$ with $x_u = 0$

Case 2: An Inhibition edge indicates negative regulatory (**Figure 8B**).

a) If $x_d = -1$: there is a predecessor node u and an edge $u \dashv d$ with $x_u = 1$;

b) If $x_d = 1$: there is a predecessor node u and an edge $u \dashv d$ with $x_u = -1$;

c) If $x_d = 0$: node u has no predecessor; or an edge $u \dashv d$ with $x_u = 0$

Case 3: Multiple activation edges connect to the same node d (**Figure 8C**).

a) If $x_d = -1$: there is at least a predecessor node u with $x_u = -1$ and the states of other predecessor nodes are un-changed (0);

b) If $x_d = 1$: there is at least a predecessor node u with $x_u = 1$ and the states of other predecessor nodes are un-changed (0);

c) If $x_d = 0$: the states of all the predecessor nodes are un-changed.

Case 4: Multiple inhibition edges connect to the same node d (**Figure 8D**).

a) If $x_d = -1$: there is at least a predecessor node u with $x_u = 1$ and the states of other predecessor nodes are no change (0);

b) If $x_d = 1$: there is at least a predecessor node u with $x_u = -1$ and the states of other predecessor nodes are no change (0);

c) If $x_d = 0$: the states of all the predecessor nodes are no change.

Case 5: Mixed reactions connect to the same node d (**Figure 8E**).

a) If $x_d = -1$: there is at least a predecessor node u has the impact to down-regulate protein d.

b) If $x_d = 1$: there is at least a predecessor node u has the impact to up-regulate protein d.

c) If $x_d = 0$: the states of all the predecessor nodes are un-changed.

Except **Case 3**-**5**, there is also a kind of special situation for **Figure 8(A-C)**: Node d may stay un-changed (0), up-regulated (1) or down-regulated (-1) if there are at least two predecessor nodes have the impact of up- and down-regulation on protein d, respectively. Here, we give three examples to elaborate this special situation:

Example A for Figure 8C: an edge $u \rightarrow d$ with $x_u = 1$ and another edge $t \rightarrow d$ with $x_t = -1$;

Example B for Figure 8D: an edge $u \dashv d$ with $x_u = 1$ and another edge $t \dashv d$ with $x_t = -1$;

Example C for Figure 8E: an edge $u \rightarrow d$ with $x_u = 1$ and another edge $t \dashv d$ with $x_t = 1$;

S2. **Computational procedure: Inference of cell-specific pathways with DILP**

Linear Programming is a novel approach for systemic modeling and network optimization[1,2]. Some previous works mainly studied the states of signaling proteins and signaling events with binary variables, however, Boolean states ("activated" or "in-activated") are not sufficient enough to represent the variations of phosphor-signals under different conditions. In this study, we proposed to develop a time-series-data-driven Integer Linear Programming (simply called as dynamic ILP or DILP) approach to infer OCs-mediated myeloma-specific signaling pathways.

The signaling pathway network is defined as a set of signaling proteins $P = \{1,2,\dots,j,\dots n_s\}$ and reactions $E = \{1,2,\dots,i,\dots n_r\}$. All of the proteins were measured at several time points, indexed by the set $T = \{t_1, t_2, \dots, t_L\}$. A discrete variable $x_{j,k} \epsilon \{-1,0,1\}$ indicates if the protein j is up-regulated ($x_{j,k} = 1$), down-regulated ($x_{j,k} = -1$), or un-changed ($x_{j,k} = 0$) at time point k. The reaction i ($i \in E$) can be represented as $u \rightarrow d$ (activation) or $u \dashv d$ (inhibition), where u and d are the upstream and downstream proteins of this reaction, respectively ($u, d \in P$). The *impact* ("positive regulatory" or "negative regulatory") of the upstream protein u on downstream protein d is described as the regulating effect from u to d when protein u is up- or down-regulated. The reaction $u \rightarrow d$ indicates

that the protein u has positive regulatory role of the protein d; Similarly, the reaction $u \dashv d$ denotes that the protein u has a negative regulatory role of protein d. When the state of protein u is un-changed, u has no impact on its downstream protein d.

The sign of edge i is denoted by r_i ($r_i = 1$ for activation and $r_i = -1$ for inhibition). The binary variable $z_{i,k}$ indicates whether the reaction i ($i \in E$) took place at time point k according to the model prediction (0 and 1 mean "take place" and "not"). Here, we also introduce the binary variables $u_{i,k}^{+}$ and $u_{i,k}^{-}$ to represent the impact of protein u (via reaction i) to up- or down-regulate protein d at time point k. Reaction i with upstream protein u has the impact of up-regulating its target protein d at time point k ($u_{i,k}^{+}$=1) if $r_i . x_{u,k} = 1$; elsewise, $u_{i,k}^{+} = 0$. Similarity, reaction i with u has the impact of down-regulating d at time point k ($u_{i,k}^{-} = 1$) if $r_i . x_{u,k} = -1$. In any other case, $u_{i,k}^{-} = 0$. The variables $u_{i,k}^{+}$ and $u_{i,k}^{-}$ can be constrained as follows:

$$u_{i,k}^{+} = \max(0, r_i . x_{u,k} - z_{i,k}) \qquad (3)$$

$$u_{i,k}^{-} = \max(0, -r_i . x_{u,k} - z_{i,k}) \qquad (4)$$

From formula (3)-(4), if the reaction i didn't take place at time point k, the protein u didn't have impact to change the downstream protein d through this reaction ($u_{i,k}^{+} = u_{i,k}^{-} = 0$). The equations (3)-(4) can be represented with linear constraints (5-14) based on Melas's work [2]:

$$u_{i,k}^{+} \geq 0 \qquad (5)$$

$$u_{i,k}^{+} \geq r_i * x_{u,k} - z_{i,k} \qquad (6)$$

$$u_{i,k}^{+} + 3d1_{i,k} \leq 3 \qquad (7)$$

$$u_{i,k}^{+} + z_{i,k} - r_i * x_{u,k} + 3d2_{i,k} \leq 3 \qquad (8)$$

$$d1_{i,k} + d2_{i,k} = 1 \qquad (9)$$

$$u_{i,k}^{-} \geq 0 \qquad (10)$$

$$u_{i,k}^{-} \geq -r_i * x_{u,k} - z_{i,k} \qquad (11)$$

$$u_{i,k}^{-} + 3d3_{i,k} \leq 3 \qquad (12)$$

$$u_{i,k}^{-} + z_{i,k} + r_i * x_{u,k} + 3d4_{i,k} \leq 3 \qquad (13)$$

$$d3_{i,k} + d4_{i,k} = 1 \tag{14}$$

Hence, based on the sign of edge i and the state of start node $x_{u,k}$ at time point k, $u_{i,k}^{+}$ and $u_{i,k}^{-}$ are calculated. When protein u is un-changed, $u_{i,k}^{+} = u_{i,k}^{-} = 0$; else wise, $u_{i,k}^{+} + u_{i,k}^{-} = 1$.

At last, the binary variables $x_{d,k}^{+}$ and $x_{d,k}^{-}$ were introduced to represent the impact (effect) for node d of being up- or down-regulated depending its parental nodes (upstream proteins) at time point k. Node d has the impact of being up-regulated ($x_{d,k}^{+} = 1$) if $u_{i,k}^{+} = 1$ and node d has the impact of being down-regulated ($x_{d,k}^{+} = 1$) if $u_{i,k}^{-} = 1$. The state ($x_{d,k}$) of node d at time point k depends on $x_{d,k}^{+}$ and $x_{d,k}^{-}$.

$$x_{d,k}^{+} \geq u_{i,k}^{+} \tag{15}$$

$$x_{d,k}^{-} \geq u_{i,k}^{-} \tag{16}$$

$$x_{d,k}^{+} \leq \sum_{\substack{w \in P, q \in E \\ u_w \xrightarrow{q} u_d}} u_{q,k}^{+} \tag{17}$$

$$x_{d,k}^{-} \leq \sum_{\substack{w \in P, q \in E \\ u_w \xrightarrow{q} u_d}} u_{q,k}^{-} \tag{18}$$

$$x_{d,k} \leq x_{d,k}^{+} \tag{19}$$

$$x_{d,k} \geq -x_{d,k}^{-} \tag{20}$$

$$x_{d,k} \leq 2x_{d,k}^{+} - x_{d,k}^{-} \tag{21}$$

$$x_{d,k} \geq -2x_{d,k}^{-} + x_{d,k}^{+} \tag{22}$$

According to equations (15)-(22), we can see node d may has the impact of being both up- and down-regulated if $x_{d,k}^{+} = x_{d,k}^{-} = 1$. The state of protein d cannot be accurately determined if $x_{d,k}^{+} = x_{d,k}^{-} = 1$. The states of a downstream protein and his all parental nodes (upstream proteins) met the ***state consistent rules*** which were detailedly described in **Supplementary Text S1**.The constraints (15-22) based on Melas's work [2].

To find an optimal set of reactions from the original generic pathway map, we have introduced the binary variable y_i and $z_{i,k}$. The variable y_i denotes 1 if the reaction i is removed in the inferred cell-specific signaling network, and 0 else wise. The variable $z_{i,k}$ denotes 0 if the reaction i ($i \in E$) takes place at the time point k, and 1 else wise. The state of $z_{i,k}$ may affect the fitting error between

experimentally measured and predicted values of proteins. Moreover, combination of formulas (23-24) reflects how the state of reaction i at time point k ($z_{i,k}$) affects the presence of this reaction in the final inferred cell-specific pathway network. Formula (23) indicates that reaction i is present in the cell-specific pathway network if it takes place at least at one time point. Formula (24) denotes that the reaction i is not included in the cell-specific pathway network if this reaction doesn't occur at all of the time points.

$$z_{i,k} \geq y_i, \quad k \in T, i \in E \tag{23}$$

$$1 - y_i \leq \sum_{k \in T} (1 - z_{i,k}) \tag{24}$$

To infer cell-specific pathway networks, we applied our DILP approach with above constraints to minimize the differences between experimentally measured and predicted values of signaling proteins, as well as to obtain a minimized sub-network of original generic pathway map by optimizing the following objective function shown in formula (25):

$$\min\{\sum_{k \in T} \sum_{j \in P} (m_{j,k} - x_{j,k})^2 + \beta \sum_{i \in E} y_i\} \tag{25}$$

In this objective function, the first term represents the fitting error between experimentally measured and predicted values and the second term denotes a set of reactions from the original generic signaling network. The measured and predicted values of j–th protein at time point k were denoted as $m_{j,k}, x_{j,k} \in \{-1,0,1\}$, respectively. When $m_{j,k}$ and $x_{j,k}$ are equal, the value of term $(m_{j,k} - x_{j,k})^2$ will be 0; otherwise it is either 1 or 4. Hence, optimization of the above objective function might induce local-optimal solution because of the non-uniform distribution of the term $(m_{j,k} - x_{j,k})^2$. In order to address this bias, binary variable $a_{j,k}$ (taking value of 0 or 1) was designed as the difference between $m_{j,k}$and $x_{j,k}$ as following constraints (26-27): $a_{j,k}$ will be 1 if $m_{j,k}$ is not equal to $x_{j,k}$; the minimum of formula (28) will automatically set $a_{j,k}$as 0 if $m_{j,k}$ is equal to $x_{j,k}$.

$$a_{j,k} \geq \frac{m_{j,k} - x_{j,k}}{2} \tag{26}$$

$$a_{j,k} \geq \frac{x_{j,k} - m_{j,k}}{2} \tag{27}$$

Then the term $(m_{j,k} - x_{j,k})^2$ in above objective function was replaced by $a_{j,k}$.Therefore, the objective function (25) can be simplified to formula (28).

$$\min\{\sum_{k\in T}\sum_{j\in P} a_{j,k} + \beta\sum_{i\in E} y_i\} \quad (28)$$

The negative constant β in formula (28) is used to obtain a minimum sub-graph of the generic pathway maps as the finalized cell-specific pathways (here, we have $-\frac{1}{|E|} < \beta < 0$), in which $|E|$ is the number of reactions in the network.

In addition, minimizing the optimal network topology by edge removals might eliminate some reactions, leading to some phosphor-signals can't be transduced into downstream proteins (such as two examples shown in **Supplementary Fig. S6** and **S7**, respectively). After obtaining a single minimized sub-graph of generic pathway network via DILP approach, we designed a strategy for searching the missing edges. The missing edges were retrieved to the optimal network obtained from formula (28) if the goodness of fit (defined in **Supplementary Text S3**) was un-changed. The details of addition of missing edges were described in **Supplementary Text S4**.

S3. Computational procedure: fitting precision of data (goodness of fit)

In the process of searching the optimal cell-specific pathways, we defined fitting precision (FP) to describe the goodness of fit between the predicted values derived by our DILP approach and the measured values of proteins under all the time points.

$$FP = \frac{\sum_{j=1}^{N}\sum_{k=1}^{T} a_{j,k}}{N \times T} \times 100\% \quad (29)$$

Where the binary variable $a_{j,k}$ indicates the difference between the measurement $m_{j,k}$ and predicted value $x_{j,k}$ of j –th protein at the time point k. N and T are the total number of measured proteins and time points, respectively. The value of fitting precision (FP) is in the range from 0% to 100%.

S4. Computational procedure: addition of missing edges into a single optimal solution

Minimizing the optimal network topology by edge removals might eliminate some reactions, leading to some of phosphor-signals can't be transduced from upstream to downstream. Here, we firstly represent **Supplementary Fig. S6**-**S7** as two examples to describe the situation which might occur in the optimization.

Supplementary Fig. S6(A-B) represent a generic pathway map and the inferred cell-specific pathway map, respectively. The states of x_A and x_C were measured as up-regulation, and x_B was unknown (un-measured). The goodness of fit reaches to 100% after optimization; however, minimizing the optimal

network topology by edge removals eliminate the reactions r_1 and r_2, which lead to the phosphor-signal from x_A can't be transduced into downstream proteins x_C. The predicted states of x_A and x_C in the network shown in **Supplementary Fig S6(B)** were up-regulated ("1") and x_b was up-changed ("0"). The reasonable inferred cell-specific pathway network should be as shown in **Supplementary Fig. S6(C).**

In **Supplementary Fig. S7(A)**, the downstream protein x_D is activated if and only if at least one of three upstream proteins x_A, x_B, and x_C is activated. Given a scenario that x_A, x_B, and x_D were measured as up-regulation, and x_C is unknown, the optimization of formula (28) might deliver one of two candidate optimal solutions shown in **Supplementary Fig. S7(B-C)**. **Supplementary Fig. S7(B-C)** indicate that two candidate optimal network topology which both have the best fitting precision (100%) to measurements. However, minimization of network topology in this case also result in one upstream real effect cannot be transduced to downstream. Therefore, we consider the solution shown in **Supplementary Fig. S7(D)** might be more reasonable which consistent with the measurement. As to the node x_C, its predicted state can be one of three possible states (up-regulation, down-regulation, and no-change) because un-measured nodes would not be included in the objective function (28).

In this study, we proposed a method to detect the missing edges whose addition would not reduce the goodness of fit. After computing a single minimal sub-graph of generic pathway network, we consider a greedy strategy for searching potential missing edges and keeping the same goodness of fit as that in the first run of formula (28). The pseudo-code for adding the missing edges was described as follows:

Step 1: Get an initial optimal solution (OS_1) from formula (28) with a negative α (minimize the topology).
Step 2: Based on OS_1:

```
For each reaction i ∈ E,
    At the time point k, x_{par,k} and x_{chd,k} are the predicted values of parent and child node
    involved in reaction i, respectively.
    If (x_{par,k}*x_{chd,k}==1) && (y_i==1)   %activation is missed
        Generate a new constraint c_i to keep z_{i,k} ≤ 0 at time point k.
        CS = {CS, c_i};   % add c_i to a constraint set CS
    End If

    If (x_{par,k}*x_{chd,k}==-1) && (y_i==1)   %inhibition is missed
        Generate a new constraint c_i to keep z_{i,k} ≤ 0 at time point k.
        CS = {CS, c_i};   % add c_i to a constraint set CS
    End If
End for
```

Step 3:

(A)Get a new solution (OS_2) from formula (28) with a negative α and the extra constraint set CS (minimize the topology). **% Add missing edges with extra constraints**

(B)If the fitting precision in OS_2 is no less than that in OS_1, go to step 4;

Otherwise, $FOS = OS_1$, go to step 6.

Step 4:

(A)Compare OS_1 and OS_2, detect all the edges d_j if y_j==1 in both OS_1 and OS_2.

(B) Delete all these edges d_j from the topology of generic pathway network (GPN)

(C) Obtain a sub-graph (SR) from GPN.

Step 5:

(A) Get the optimal solution (OS_3) from formula (28) with a positive α and network topology SR. (maximize the topology on the sub-network scale).

(B) If the fitting precision in OS_3 is no less than that inOS_1, $FOS = OS_3$, go to step 6.

Step 6: Exit.

Here, we still took **Supplementary Fig. S7(A)** as an example to illustrate our greedy search strategy for searching and adding the missing edges. At first, we might get a solution such as **Supplementary Fig. S7(B)** and the edge $x_B \rightarrow x_D$ was a missing edge. After step 2, an extra constraint set for $x_B \rightarrow x_D$ was added and went into step 3. And then we obtained another optimal solution shown in **Supplementary Fig. S7(C)** which also induced the same fitting precision as the solution in **Fig. S7(B)**. Hence, we consider two cases in **Figure S7(B-C)** are both the candidate solutions; however, they both missed an edge. In step 4, our approach found the link from $x_C \rightarrow x_D$ can be removed because x_C is un-measured and a minimal sub-graph of the generic pathway network is required. After step 4, a subset of reactions SR was obtained which eliminated the link $x_C \rightarrow x_D$. Last step, we consider the required optimal solution will restrict in the scope of SR, hence, we run the optimization with formula (28) again to search a maximal topology with the same fitting precision and covered the missing ages. Finally, we got an adjusted solution shown in **Supplementary Fig. S7(D)**, which also have the same goodness of fit (100%) as **Supplementary Fig. S7 (B-C)**. Similarity, **Supplementary Fig. S6(C)** represents that the addition of two reactions (r_1 and r_2) still keep the goodness of fit as 100%.

S5. Computational procedure: prediction of drug treatment effects by state transition analysis

Based on the established network topological structure and transfer functions (Boolean operation), state transition analysis in *Boolean networks* is a kind of approach to predicting the future state of each node from the current states of its parental nodes [3-5]. The assumption of state transition is that: the state of protein j at time point $k+1$ ($x_{j,k+1}$) is associated with the states of its parental proteins at time point k [5,6]. Thus, we predicted the state of pathway network at time point $k+1$ from the state at time

point k through state transition, following perturbation of the cell-specific pathway network with drugs. Given the inferred cell-specific pathway network topology G and the states of all the proteins involved in the network at time point k: $X_k = [x_{1,k}, x_{2,k}, \dots, x_{n,k}]$ (n is the total number of proteins in the network), we can obtain the states of these proteins at time point $k+1$ using the following formula:

$$X_{k+1} = F(X_k, G) \quad (30)$$

where $k = 1,2,\dots,L$, and $x_{j,k} \epsilon \{-1,0,1\}$. F is a set of transfer functions to change the signaling network from one state to another. In Boolean networks, transfer functions are denoted by using logical expressions via Boolean operators [5,7]. However, it is difficult to represent the transfer functions using an established mathematic expression, if each signaling protein has three possible states (1, -1, or 0) [2]. In our study, the transfer functions F were represented by a set of integer linear constraints.

Let's use $X_0 = \{x_{1,0}, x_{2,0}, \dots, x_{j,0}, \dots, x_{n,0}\}$ ($x_{j,0} \in \{-1,1,0\}$) to denote a measured state of signaling network without any intervention or treatment. When cells are treated by an inhibitor, we assume protein j is targeted by this inhibitor, and then the state of above signaling network after treatment is changed as $X_0 = \{x_{1,0}, x_{2,0}, \dots, -1, \dots, x_{n,0}\}$. We used X_0 as the initial state to predict the performance of the inhibition or perturbation. We then used formula (30) to calculate the next state (X_1) of X_0 and repeat this process until the signaling network reached to steady state [7]. We eventually generated a set of states $\{X_1, X_2, \dots, X_A\}$. X_A is regarded as the finally effects of drug treatment in cell-specific pathway network.

In here, the constraints involved in the transfer functions F were represented as following constraints:

$$u^{+}_{i,k\to k+1} \geq 0 \quad (31)$$

$$u^{+}_{i,k\to k+1} \geq r_i * x_{u,k} \quad (32)$$

$$u^{+}_{i,k\to k+1} + 3d1_{i,k} \leq 3 \quad (33)$$

$$u^{+}_{i,k\to k+1} - r_i * x_{u,k} + 3d2_{i,k} \leq 3 \quad (34)$$

$$d1_{i,k} + d2_{i,k} = 1 \quad (35)$$

$$u^{-}_{i,k\to k+1} \geq 0 \quad (36)$$

$$u^{-}_{i,k\to k+1} \geq -r_i * x_{u,k} \quad (37)$$

$$u^{-}_{i,k\to k+1} + 3d3_{i,k} \leq 3 \quad (38)$$

$$u^{-}_{i,k\to k+1} + r_i * x_{u,k} + 3d4_{i,k} \leq 3 \quad (39)$$

$$d3_{i,k} + d4_{i,k} = 1 \quad (40)$$

$$x^+_{d,k+1} \geq u^+_{i,k\to k+1} \quad (41)$$

$$x^-_{d,k+1} \geq u^-_{i,k\to k+1} \quad (42)$$

$$x^+_{d,k+1} \leq \sum_{\substack{w\in P, q\in E \\ u_w \xrightarrow{q} u_d}} u^+_{q,k\to k+1} \quad (43)$$

$$x^-_{d,k+1} \leq \sum_{\substack{w\in P, q\in E \\ u_w \xrightarrow{q} u_d}} u^-_{q,k\to k+1} \quad (44)$$

$$x_{d,k+1} \leq x^+_{d,k\to k+1} \quad (45)$$

$$x_{d,k+1} \geq -x^-_{d,k\to k+1} \quad (46)$$

$$x_{d,k+1} \leq 2x^+_{d,k\to k+1} - x^-_{d,k\to k+1} \quad (47)$$

$$x_{d,k+1} \geq -2x^-_{d,k\to k+1} + x^+_{d,k\to k+1} \quad (48)$$

Similar as **Supplementary Text S3**, the binary variables $u^+_{i,k\to k+1}$ and $u^-_{i,k\to k+1}$ represent the impact of protein u (via reaction i) at time point k to up- or down-regulate the protein d at time point $k+1$. Reaction i with upstream protein u has the impact of up-regulating its target protein d at time point k ($u^+_{i,k\to k+1}$ =1) if $r_i . x_{u,k} = 1$; elsewise, $u^+_{i,k\to k+1} = 0$. Similarity, reaction i with u has the impact of down-regulating d at time point k ($u^-_{i,k} = 1$) if $r_i . x_{u,k} = -1$. In any other case, $u^-_{i,k\to k+1} = 0$. The value of $u^+_{i,k\to k+1}$ and $u^-_{i,k\to k+1}$ are calculated through above Constraints (31-40). Similarly, two binary variables $x^+_{d,k\to k+1}$ and $x^-_{d,k\to k+1}$ are introduced to represent the impact for node d of being up- or down-regulated at time point $k+1$ depending on the activity of its upstream edges at time point k. Protein d has the impact of being up-regulated ($x^+_{d,k\to k+1} = 1$) if $u^+_{i,k\to k+1} = 1$ and node d has the impact of being down-regulated ($x^-_{d,k\to k+1} = 1$) if $u^-_{i,k\to k+1} = 1$. Constraints (41-48) denote that the state of protein d at time point $k+1$ ($x_{d,k+1}$) was determined by variable $x^+_{d,k\to k+1}$ and $x^-_{d,k\to k+1}$.

The state of protein d at time point $k+1$ cannot be accurately determined if there are at least two predecessor nodes (upstream proteins) have the impacts of up- and down-regulation on protein d ($x^+_{d,k\to k+1} = x^-_{d,k\to k+1} = 1$). We have strategies to address two types of cases: (1) If one of the upstream nodes of protein d is a drug target, and the state of protein d at time point k was up-regulated ($x_{d,k} = 1$), then we set $x_{d,k+1} = -1$ to indicate the immediate effect of this drug on the target. (2) If none of the upstream nodes of protein d is a drug target, we set $x_{d,k+1} = x_{d,k}$.

Supplementary Figures

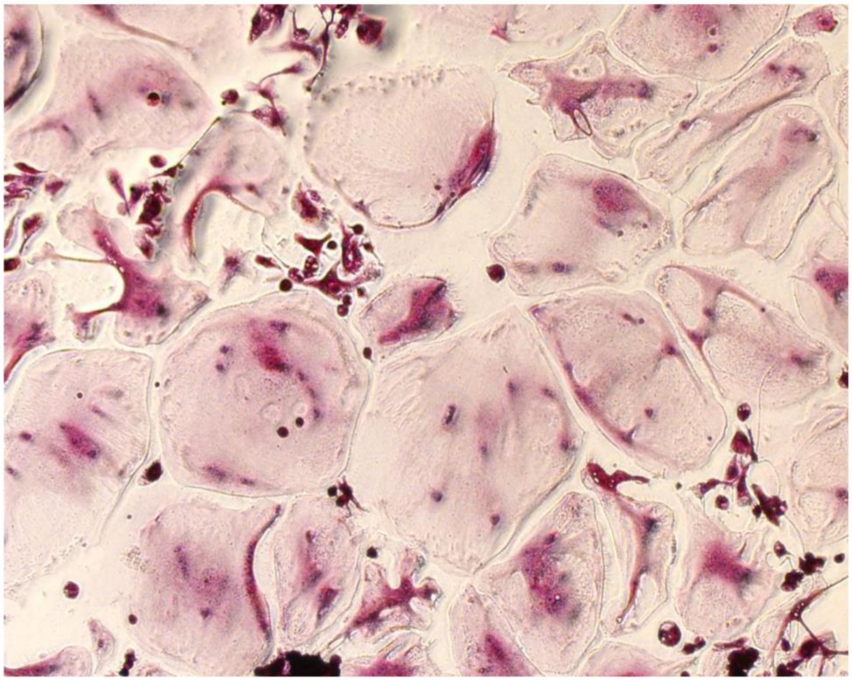

Fig. S1 TRAP staining of OCs (20× amplification).

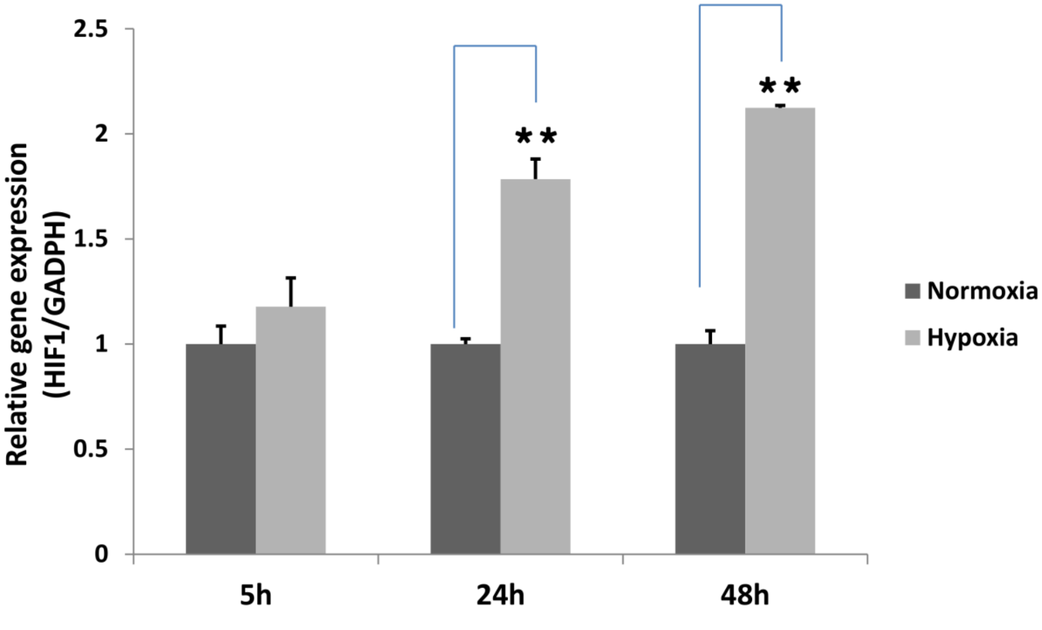

Fig. S2 HIF1 expression in myeloma cells under hypoxic condition.

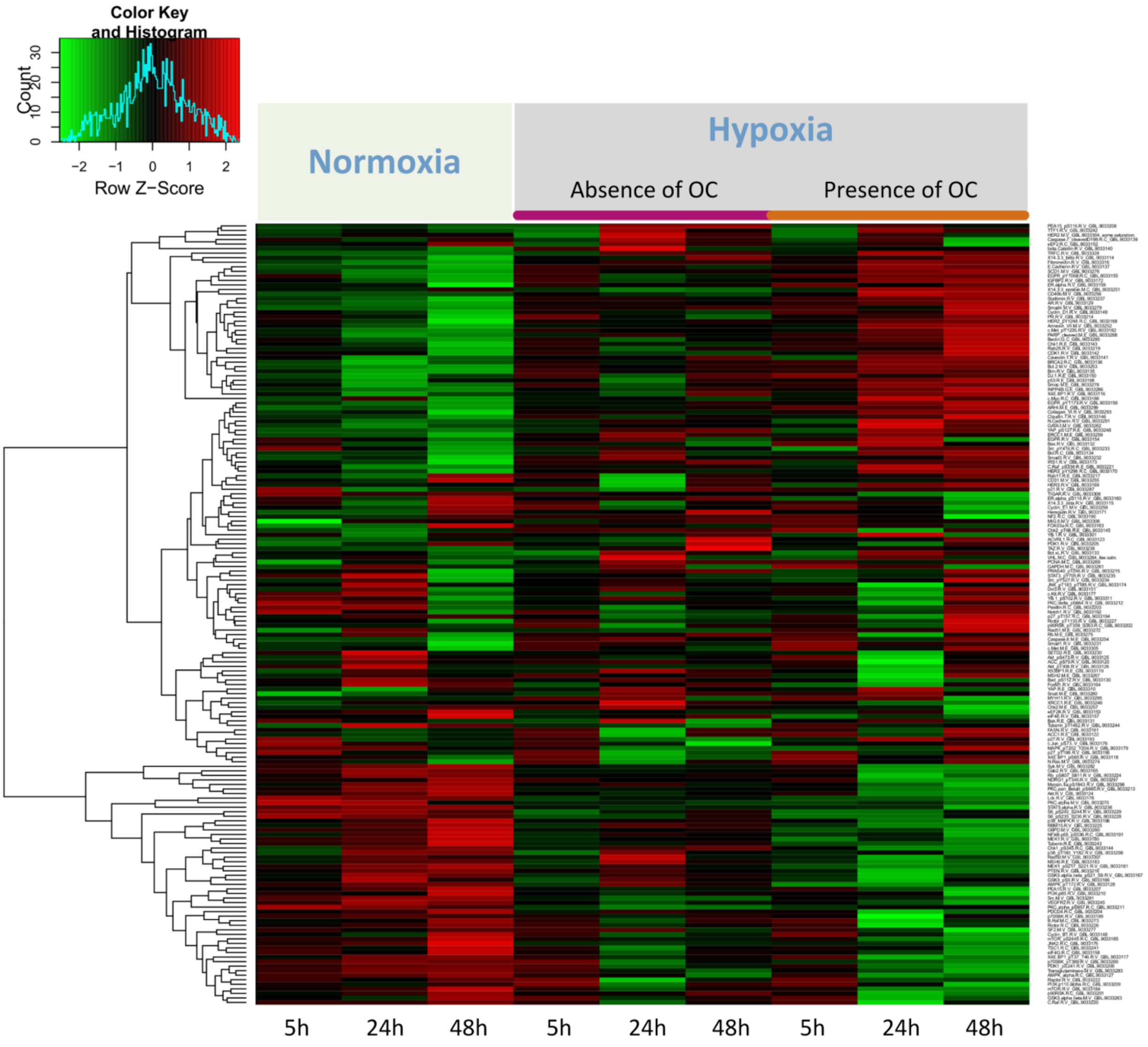

Fig. S3 The heatmap of all the RPPA proteins in both conditions.

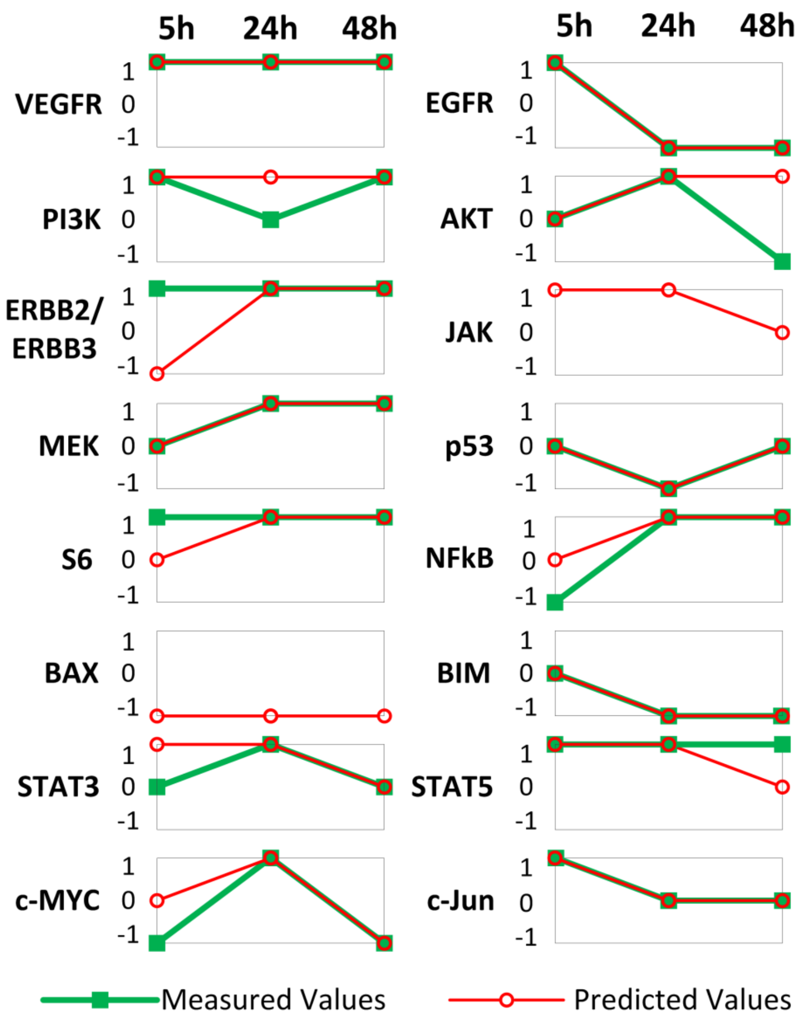

Fig. S4 The predicted states and measured states of some key factors in the inferred specific pathways of MM cells in normoxic condition. The integer value "-1", "1", and "0" are represented as down-regulation, up-regulation, and no-change, respectively.

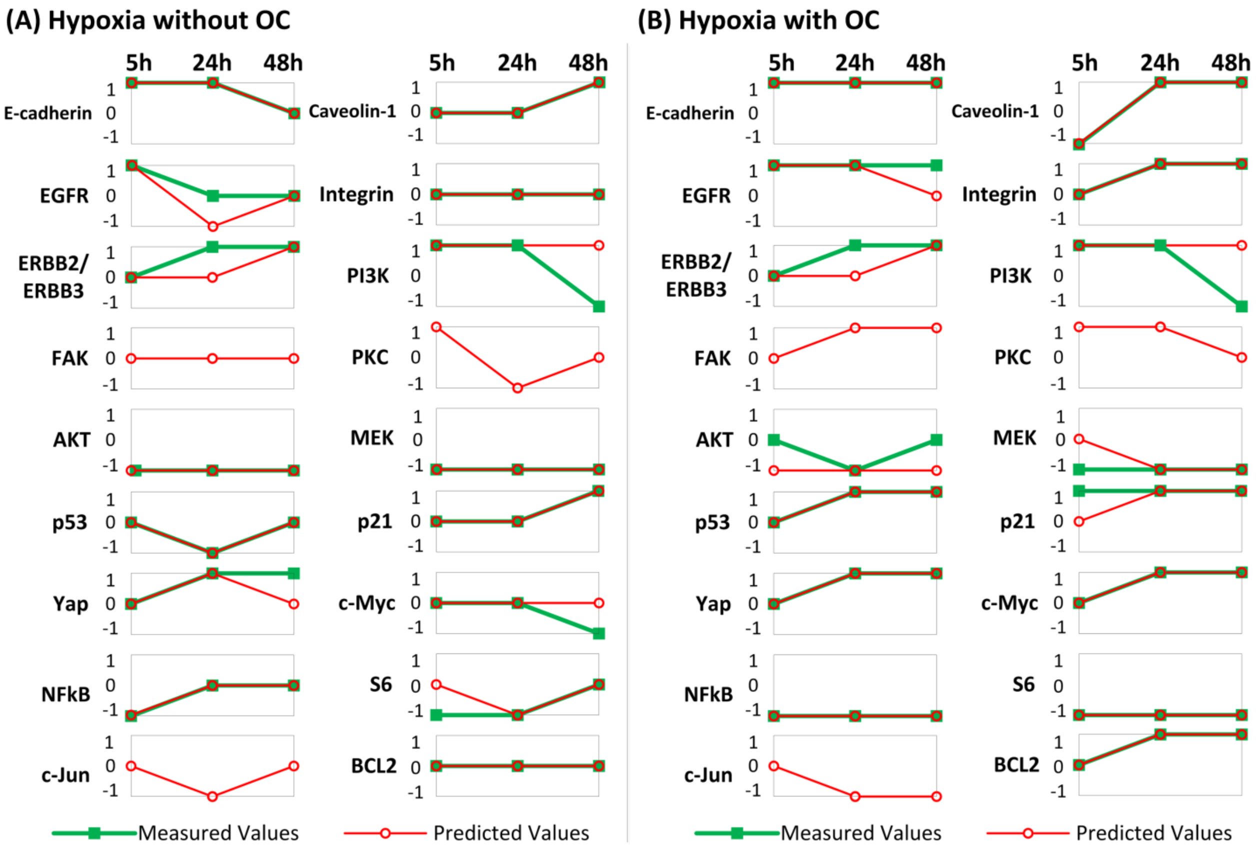

Fig. S5 The predicted states and measured states of key factors in the inferred specific pathways of MM cells in hypoxic condition with the presence versus absence of OC. (A) The states of some important proteins in the MM-specific pathways in hypoxia without OC. (B) The states of some important proteins in the MM-specific pathways in hypoxia with OC. The integer value "-1", "1", and "0" are represented as down-regulation, up-regulation, and no-change, respectively.

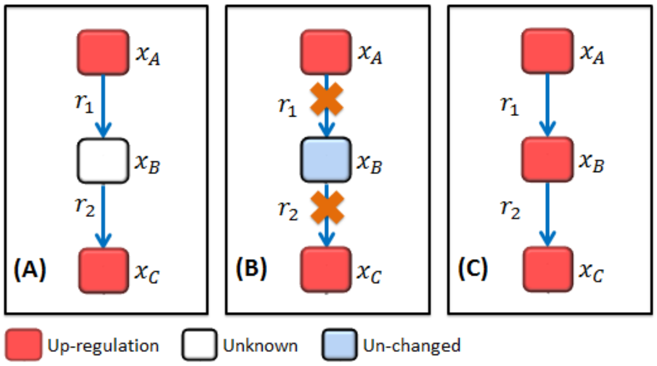

Fig. S6 An example about the addition of missing edges. (A) A generic pathway map; (B) An inferred cell-specific pathway map. The states of x_A and x_C were measured as up-regulation, and x_B was unknown (un-measured). The goodness of fit reaches to 100% after optimization; however, minimizing the signaling network topology by edge removals will eliminate the reactions r_1 and r_2, which lead to the phosphor-signal from x_A can't be transduced into downstream proteins x_C. (C) The optimal cell-specific pathway map after addition of missing edges.

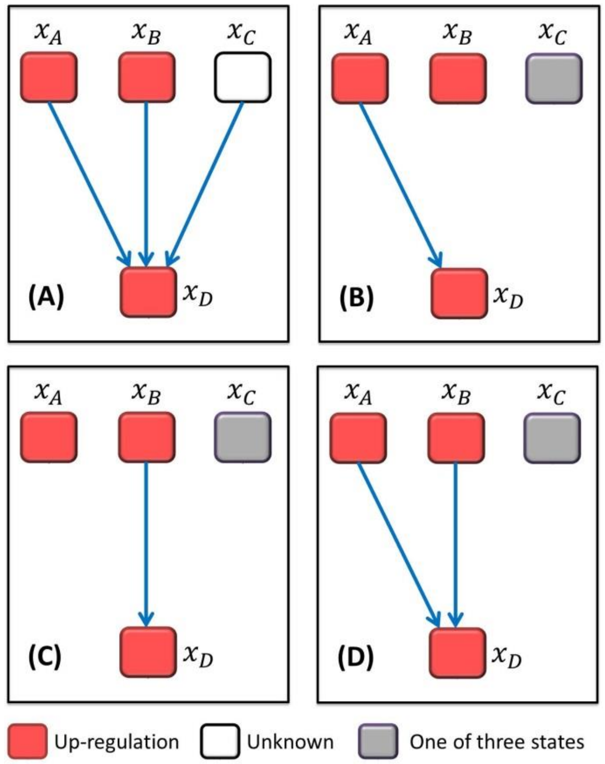

Fig. S7 Another example about the addition of missing edges. (A). a generic pathway map and the measurements of three nodes. (B-C) Two candidate cell-specific pathway networks. These two optimal solutions obtained from our model by minimizing both the fitting error and network topology. (D) the final optimal cell-specific pathway map after addition of missing edges.

Supplementary Tables

Table S1. The fitting precision of the proposed DILP model on the processed RPPA data**.**

	**Normoxia** (MM+OC VS. MM)	**Hypoxia (CTL)** (Hyp MM VS. Nor MM)	**Hypoxia (OC)** (Hyp MM+OC VS. Nor MM+OC)
**5H**	79.17%	92%	84%
**24H**	87.50%	88%	96%
**48H**	87.46%	88%	88%
**Avg.**	84.72%	89.33%	83.33%

Table S2. The expressions of all the proteins involved in the OC-mediated MM-specific pathways in normoxia (with the presence of OC) before and after treatment with PI3K inhibitor. The expressions of all the proteins before treatment (the second column in **Table S2**) were collected (at 24h) after we inferred the MM-specific pathways by fitting the measured data with the generic pathway map (see **Fig. S4**). The expressions of all the proteins after treatment (the third column in **Table S2**) were predicted by the state transition analysis with our DILP approach. In normoxia, treatment on myeloma cells with Pi3K inhibitor induced the changes of some key proteins (orange color).

Proteins	Before Treatment	After Treatment
Akt	1	-1
p53	-1	1
Bim	-1	1
cJun	0	-1
cMyc	1	-1
Caspase8	-1	-1
EGFR	-1	-1
JNK	0	-1
MEK	1	-1
mTOR	1	-1
NFkB	1	-1
p38	1	-1
p70S6K	1	-1
PDK1	1	-1
PI3K	1	-1
PKC	1	-1
S6	1	-1
STAT5	1	-1
VEGFR	1	1
ERBB2	1	1
Src	1	-1
STAT3	1	-1
JAK	1	-1
Raf	1	-1
MKK	-1	-1
ERK	1	-1
FOXO3	-1	1
BID	-1	-1
BAX	-1	-1

Table S3. The expressions of all the proteins involved in the OC-mediated MM-specific pathways in hypoxia (with the presence of OC) before and after treatment with combination of PI3K and integrin inhibitors. The expressions of all the proteins before treatment (the second column in **Table S3**) were collected (at 24h) after we inferred the MM-specific pathways by fitting the measured data with the generic pathway map (see **Fig. S5**). The expressions of all the proteins after treatment (the third column in **Table S3**) were predicted by the state transition analysis with our DILP approach. In hypoxia, treatment on Myeloma cells with Pi3K inhibitor induced the changes of some key proteins (orange color).

**Proteins**	**Before Treatment**	**After Treatment**
Akt	-1	-1
ERBB2	0	0
Bad	-1	-1
Bcl2	1	1
Fibronectin	1	1
cMyc	1	-1
raf	1	-1
Integrin	1	-1
Src	0	-1
EGFR	1	1
Caveolin	1	1
Collagen	1	-1
JNK	-1	-1
MEK1	-1	-1
mTOR	-1	-1
NFkB	-1	-1
p21	1	1
p38	-1	-1
p53	1	1
p70S6K	-1	-1
ECadherin	1	1
PI3K	1	-1
S6	-1	-1
B_Catenin	1	1
YAP	1	1
FAK	1	-1
PKC	1	-1
PDK1	1	-1
MKK	1	1
ERK	-1	-1
cJun	-1	-1

Table S4. The details of the antibodies used in Western blot.

Antibodies	Company	Dilution
p-AKT	Cell signaling	1:1000
p-MEK	Cell signaling	1:1000
p-Erk	Cell signaling	1:1000
c-MYC	Cell signaling	1:1000
p-FAK	Cell signaling	1:1000
P53	Cell signaling	1:1000
Fibronectin	Santa Cruz	1:1000
β-actin	Cell signaling	1:5000

References

1. Knapp, B. & Kaderali, L. Reconstruction of Cellular Signal Transduction Networks Using Perturbation Assays and Linear Programming. *Plos One* **8**(2013).
2. Melas, I.N., Samaga, R., Alexopoulos, L.G. & Klamt, S. Detecting and Removing Inconsistencies between Experimental Data and Signaling Network Topologies Using Integer Linear Programming on Interaction Graphs. *Plos Computational Biology* **9**(2013).
3. Saadatpour, A. & Albert, R. Discrete dynamic modeling of signal transduction networks. *Methods Mol Biol* **880**, 255-72 (2012).
4. Cheng, D.Z. & Qi, H.S. State-Space Analysis of Boolean Networks. *Ieee Transactions on Neural Networks* **21**, 584-594 (2010).
5. Chaves, M., Albert, R. & Sontag, E.D. Robustness and fragility of Boolean models for genetic regulatory networks. *J Theor Biol* **235**, 431-49 (2005).
6. Zhou, X. *et al.* A Bayesian connectivity-based approach to constructing probabilistic gene regulatory networks. *Bioinformatics* **20**, 2918-27 (2004).
7. Albert, R. & Wang, R.S. Discrete dynamic modeling of cellular signaling networks. *Methods Enzymol* **467**, 281-306 (2009).
